# A GWAS–machine learning framework reveals protein-synthesis pathway signals for yield in *Theobroma cacao* after population-structure correction

**DOI:** 10.1038/s41598-026-42273-w

**Published:** 2026-03-17

**Authors:** Insuck Baek, Jishnu Bhatt, Seunghyun Lim, Dongho Lee, Jae Hee Jang, Stephen P. Cohen, Amelia H. Lovelace, Moon S. Kim, Lyndel W. Meinhardt, Sunchung Park, Ezekiel Ahn

**Affiliations:** 1 Environmental Microbial and Food Safety Laboratory, Agricultural Research Service, Department of Agriculture, Beltsville, 20705 MD USA; 2 Sustainable Perennial Crops Laboratory, Agricultural Research Service, Department of Agriculture, Beltsville, 20705 MD USA; 3 Soybean Genomics & Improvement Laboratory, Agricultural Research Service, Department of Agriculture, Beltsville, 20705 MD USA

**Keywords:** *Theobroma cacao*, Yield, GWAS, Machine learning, Protein synthesis, Genomic prediction, Population structure, Plant breeding, Ribosome, Computational biology and bioinformatics, Genetics, Plant sciences

## Abstract

Improving cacao yield, a key objective in post-domestication crop improvement, remains a primary goal for breeders, but progress is often hindered by the confounding effects of population structure. To overcome this, we analyzed 346 diverse cacao accessions using an ML-based association mapping framework (with and without population structure adjustment) and a phenotype-only ML prediction of yield. By correcting for population structure, our Bootstrap Forest-based GWAS produced SNP-importance rankings whose downstream functional summaries were enriched for ribosome/translation-related terms, and several top-ranked SNPs recurred across multiple yield components (e.g., pod index and seed number) in this panel. In parallel, Neural Networks were utilized to identify cotyledon mass and length as the most powerful predictors for total wet bean mass, providing a phenotype-only prediction example for this panel. Collectively, this study provides an ML-guided, low-density association workflow and a phenotype-only prediction example for this cacao panel, while explicitly outlining limitations related to marker density and phenotype provenance.

**Supplementary Information:**

The online version contains supplementary material available at 10.1038/s41598-026-42273-w.

## Introduction

Improving the genetic potential of high-value horticultural crops is a central goal of modern agricultural science. *Theobroma cacao* L., the source of cocoa beans, is a globally important crop that underpins a multi-billion-dollar chocolate industry and supports the livelihoods of millions of smallholder farmers^1,2^. These smallholders are responsible for approximately 80% of global cocoa production^3^. Originating in the upper Amazon basin^4^, cacao exhibits rich genetic diversity, contributing to a wide range of flavors, aromas, and other desirable traits^5^. However, cacao production faces increasing challenges from emerging diseases and pests, and a growing global demand for chocolate^6,7^. These challenges necessitate the development of improved cacao varieties with enhanced yield potential, disease resistance, and environmental resilience, while maintaining and enhancing quality attributes. This continuous drive for crop improvement, from early domestication to modern breeding, requires a deep understanding of the genetic basis of key agronomic traits.

Efforts to understand genetic diversity and identify genes controlling important traits from the cacao genome have been ongoing for decades. Studies have indicated that genetic differentiation within cacao lineages results from spatial variation in selection pressures, genetic drift, or a combination of both processes^8^. Yield stability over time is a critical trait in cacao breeding^3^, yet studies focusing on temporal stability remain limited^9^. A study evaluating 34 cacao hybrid families over six years in Costa Rica revealed substantial variability in annual yield, with coefficient of variation values ranging from 113% to 642% for different years, particularly notable in years two and three, when 80% of trees produced no pods^9^. Cacao yield was contingent on interrelated factors such as pod health and disease incidence, shown through a positive correlation between the number of healthy pods and overall yield efficiency^3^. When low-yield variability is optimized for in breeding programs, newly developed cultivars are susceptible to reduced yield efficiencies, necessitating broadly informed selection of genetic traits to sustain productivity over time^3^. Genetic factors influence pod development and quality traits^10^. The selection of progeny based on pod index, calculated as the number of pods required to produce one kilogram of dried cocoa^11^, and weight can improve overall tree productivity and efficiency in production processes^10^. Monitoring genetic identity is essential as progenies from mislabeled or pollen-contaminated clones confound breeding efforts towards the development of superior varieties^12^.

Traditional breeding methods, relying on phenotypic selection and controlled crosses, have contributed to improvements in cacao varieties^13,14^. However, these methods are unable to take into account the complex interplay and inheritance of many traits^15^. The advent of genomic tools, such as genome-wide association studies (GWAS), offers new opportunities to understand inherited trait complexity and accelerate cacao improvement^16,17^. Previous studies in cacao have employed GWAS to identify loci associated with disease resistance, yield components, and bean quality attributes^11,16,17^. Complementary to association-based approaches, linkage-based QTL mapping in segregating cacao populations has identified genomic regions influencing yield components using dense SNP linkage maps and haplotype analyses, with candidate genes frequently implicating source-to-sink transport and seed/bean filling processes^18^. These QTL-based results underscore both the polygenic architecture of yield components and the value of well-powered, structured mapping populations for localizing yield-associated regions in cacao^18^. Despite the use of standard statistical corrections in association studies, strong population structure in cacao can still confound marker–trait signals and inflate broad ‘vigor’-linked associations rather than trait-specific loci. Furthermore, translating these genetic findings into practical tools for breeders remains a significant hurdle. Consequently, a comprehensive understanding of the genetic architecture of complex traits in cacao, such as those related to yield potential, has remained elusive. Only a few studies have integrated advanced machine learning techniques to enhance the prediction and understanding of complex traits in cacao, primarily using inputs such as image analysis for bean quality and canopy architecture^19,20^, sensor data for fermentation level^21^, or agronomic and climate data for yield potential^22^. However, studies that directly combine machine learning frameworks with high-throughput genomic data for trait discovery and prediction remain rare.

This study addresses these limitations by leveraging a large and diverse collection of 346 cacao accessions, utilizing publicly available phenotypic and genotypic data from the ICGT Cacao Germplasm Database^11^. These accessions, conserved *ex-situ* at the International Cocoa Genebank, Trinidad (ICGT), represent a broad range of genetic and phenotypic variation^11^. We employed a multifaceted approach, combining detailed phenotypic evaluations of 27 traits, encompassing flower, fruit, and seed characteristics, with genome-wide SNP genotyping, phylogenetic analysis, and advanced statistical methods. Specifically, we used fixed vs. unfixed analyses as a panel-specific sensitivity comparison to illustrate how population structure can alter SNP-importance rankings and downstream functional summaries^23^. Furthermore, based on various phenotypic data, we trained a machine-learning model to predict wet bean mass, a direct indicator of yield potential.

The primary objectives of this study were to: (1) investigate the relationship between genetic relatedness and phenotypic variation in a diverse collection of cacao accessions; (2) identify genomic regions and potential candidate genes associated with key traits using a Bootstrap Forest-based GWAS approach; (3) explore the relationships among traits through hierarchical clustering; and (4) develop and evaluate a machine learning model for predicting wet bean mass. We tested several key hypotheses: first, that phenotypic variation in this diverse collection would be complex and only partially explained by broad phylogenetic relationships; second, that our GWAS and machine learning models would prioritize candidate loci and summarize recurrent functional themes, and evaluate phenotype-only prediction performance under cross-validation, respectively; and finally, that population-structure adjustment would change SNP-importance rankings and downstream functional summaries in this structured panel.

## Results

### Correlation between patristic distances and phenotypic traits

To assess whether more distantly related accessions differ more in phenotype, we computed Mantel correlations between patristic distances (from the phylogeny) and phenotypic distance matrices across the 27 underlying traits (with categorical traits encoded as indicator variables where applicable) among accessions with both genotype and phenotype records for the Mantel analyses (intersection *n* = 342). Correlations were generally small (median *r* ≈ 0.10). After false discovery rate (FDR) correction (*q* < 0.05), 12 traits were FDR-significant, led by pod index (*r* = 0.25, *q* = 0.0019) and cotyledon width (*r* = 0.20, *q* = 0.0019) (Fig. S1).

### Visualization of phenotypic and genotypic diversity using PCA

**Fig. 1:**
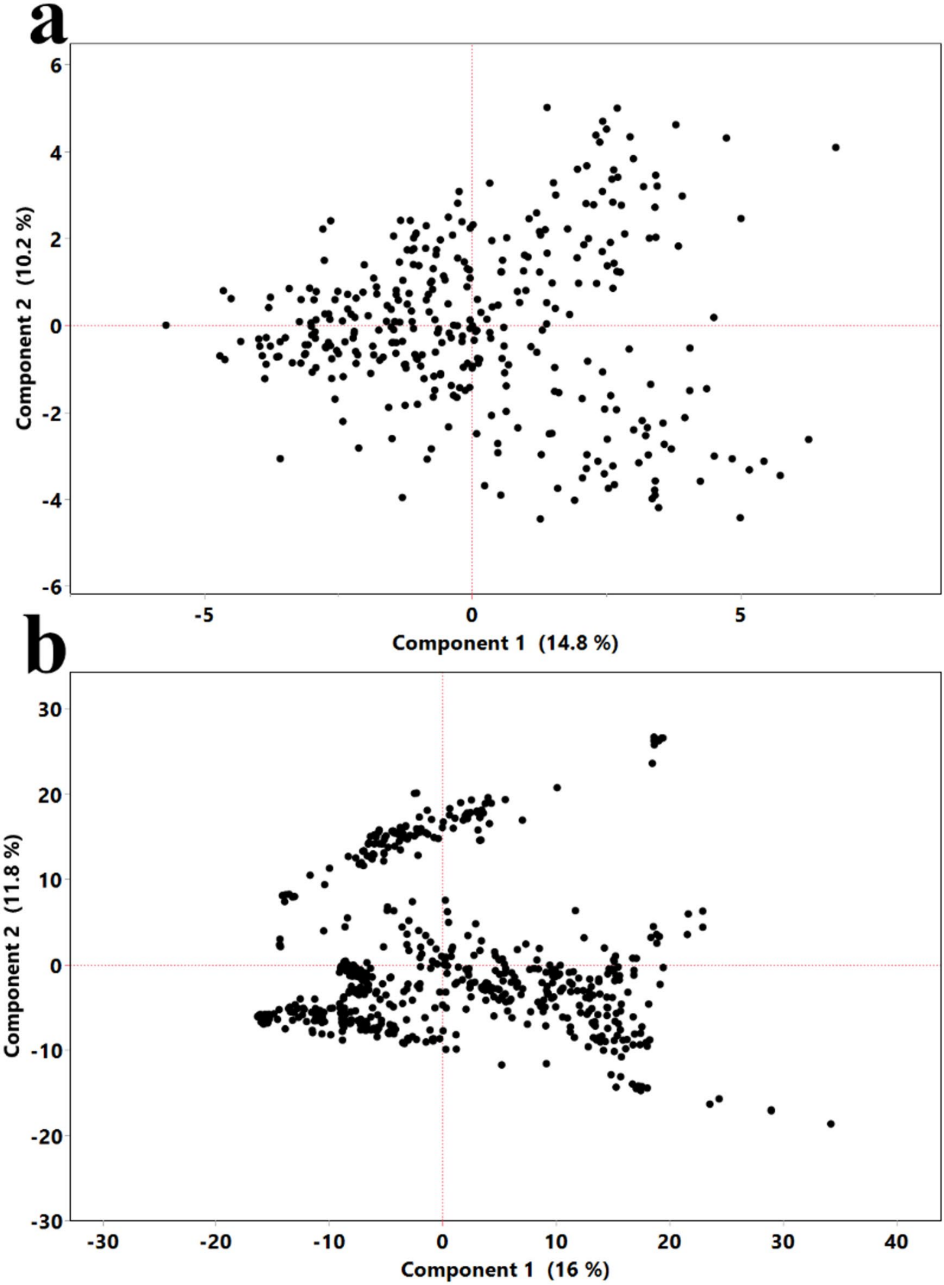
Principal component analysis of phenotypic and genotypic diversity in the cacao collection. Each point represents a cacao accession. (**a**) PCA based on phenotypic traits; PC1 and PC2 explained 14.8% and 10.2% of the variance, respectively. (**b**) PCA based on SNP genotypes; PC1 and PC2 explained 16.0% and 11.8% of the variance, respectively (PC1–PC2 shown). For the genotype PCA, PC3 explained 9.4% of the variance.

Principal Component Analysis (PCA) was used to visualize and compare the patterns of variation within the phenotypic and genotypic datasets (Fig. 1).

The PCA plot based on the phenotypic data reveals a single, diffuse cloud of points, indicating that the measured traits vary continuously across the cacao collection (Fig. 1a). The first two principal components explained 14.8% and 10.2% of the total phenotypic variation, respectively. This lack of distinct clustering suggests that no clear-cut “types” of trees can be delineated based on their physical traits alone. In contrast, the PCA based on the genotypic SNP data shows clear evidence of population structure (Fig. 1b). The accessions are organized into several distinct clusters, demonstrating that the collection is composed of different underlying ancestral groups. The first two principal components for the genotype data explained 16% and 11.8% of the total genetic variation. This visual evidence of genetic structuring compared to the continuous phenotypic variation powerfully justifies the necessity of correcting for population structure in the subsequent GWAS to avoid spurious findings.

### Genome-wide association study of key yield and morphological traits

To identify genomic regions associated with phenotypic variation, we employed a machine learning-based GWAS approach using Bootstrap Forest models. To assess the impact of population structure within the collection, we conducted two parallel analyses: a naïve model using raw phenotypes and a structure-adjusted model using ancestry-corrected residual phenotypes. The naïve model provides an exploratory view of SNP-importance rankings that may include ancestry-linked effects. In contrast, the structure-adjusted model prioritizes candidate loci whose SNP-importance rankings are less sensitive to ancestry-linked structure in this panel. The findings presented below focus on the structure-adjusted analysis; given marker density and phenotype provenance constraints, we interpret these outputs as hypothesis-generating candidate prioritization rather than definitive causal targets.

**Fig. 2:**
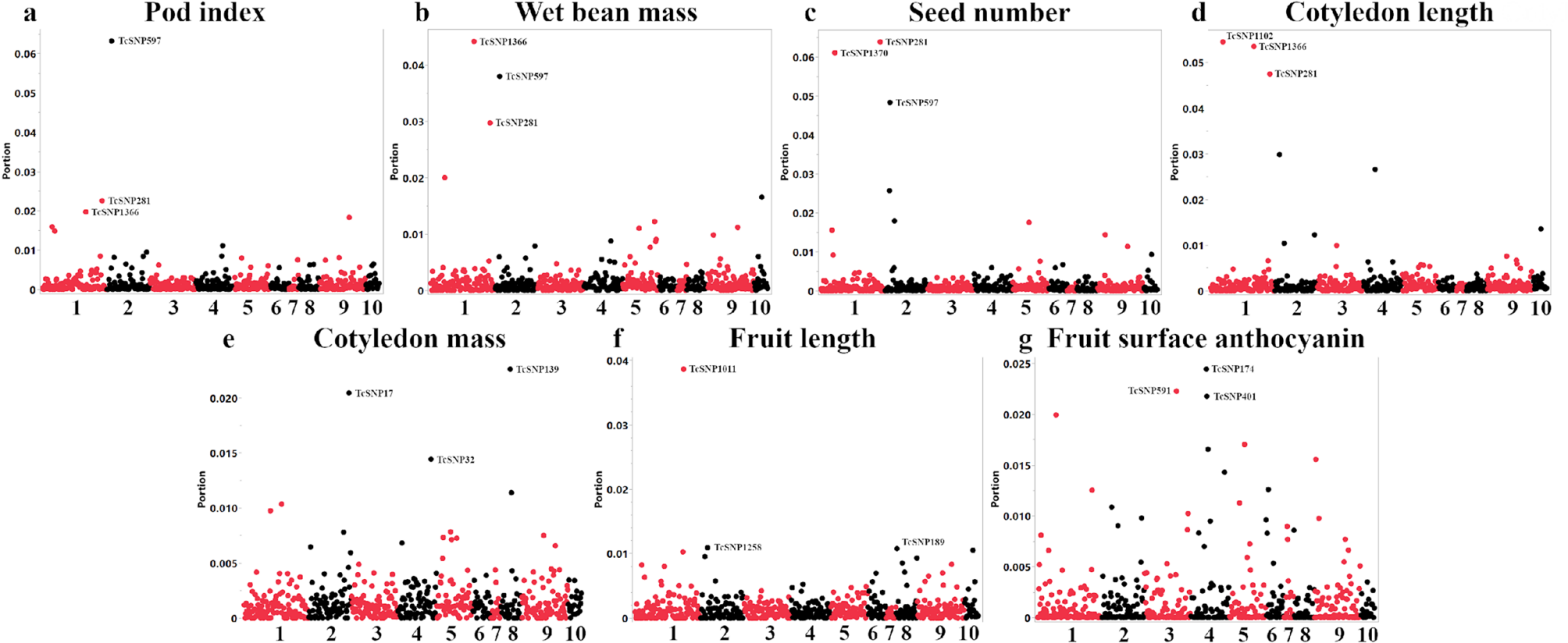
Manhattan-style plots for key traits from the structure-adjusted GWAS. Each panel displays the results from the ancestry-adjusted Bootstrap Forest GWAS for a specific key trait: (**a**) Pod index, (**b**) Wet bean mass, (**c**) Seed number, (**d**) Cotyledon length, (**e**) Cotyledon mass, (**f**) Fruit length, and (**g**) Fruit surface anthocyanin. The x-axis shows the SNP position across the 10 cacao chromosomes, while the y-axis represents the importance score (Portion) from the model, where higher values indicate greater importance in the Bootstrap Forest model.

The analysis identified multiple high-importance SNP–trait pairs in the Bootstrap Forest model. The top-ranking SNPs for seven key traits related to yield, morphology, and quality are summarized in Table 1; Fig. 2. A list of high-importance SNP–trait pairs (Portion > 0.005; reporting threshold) for all 27 traits is available in Supplementary Data 1.

### Genetic loci for yield and its core components

Across pod index, wet bean mass, and seed number, several top-ranked loci recurred in the structure-adjusted outputs, consistent with partially shared genetic influences on correlated yield components in this dataset. Notably, the SNPs TcSNP597 (encoding a 21 kDa seed protein) and TcSNP281 (encoding a 60 S ribosomal protein L31) appeared as top associations for all three of the aforementioned key yield traits. Furthermore, TcSNP1366 (encoding a Heparan-alpha-glucosaminide N-acetyltransferase) was a top locus for both pod index and wet bean mass. Collectively, these candidate summaries implicate genes annotated to ribosomal proteins, seed storage/defense, and metabolism among prioritized loci for yield components; given sparse marker density and phenotype provenance constraints, we treat these as hypothesis-generating signals rather than causal evidence.

### Associations for key predictors of yield and fruit morphology

To summarize candidate loci associated with fruit and seed morphology in the structure-adjusted analysis, GWAS also identified loci controlling important fruit and seed characteristics that contribute to overall yield potential, such as cotyledon and fruit size. For cotyledon mass, top SNPs included TcSNP139 (within the gene for a Putative Cytochrome B5 isoform D) and TcSNP17 (within a gene for a RING/U-box superfamily protein). For cotyledon length, the top-ranked SNP was TcSNP1102, within the gene for a mitochondrial cytochrome c oxidase subunit. Together, these results suggest that the size and mass of cotyledons are influenced by genes involved in cellular respiration, energy metabolism, and protein regulation. Additionally, for fruit length, a key component of pod size, the top SNP was TcSNP1011, located within a gene encoding an aspartyl protease family protein. Plant proteases are known to be involved in tissue remodeling, making this a strong candidate gene for influencing fruit development.

### Internal validation via pigmentation

For fruit surface anthocyanin level, the top-ranked SNP was TcSNP174. This SNP is located within the gene encoding 6-phosphogluconate dehydrogenase. This enzyme participates in the oxidative pentose phosphate pathway, which contributes reducing power and precursor supply for downstream biosynthetic processes, including pathways that feed into flavonoid/anthocyanin production; we present this as pathway-consistent candidate recovery rather than proof of causality.

**Table 1:** Putative candidate genes near top SNPs for key cacao traits from the structure-adjusted GWAS. Summary of the top-ranked SNPs (by Portion importance) identified by the Bootstrap Forest models after population-structure adjustment. For each SNP, the table reports chromosome (Chr; when available in the source tables), the nearest annotated gene, and the Portion importance score.

Trait	SNP ID	Chr	Putative Gene	Importance Score (Portion)
Pod index	TcSNP597	2	*Tc02v2_p004390* 21 kDa seed protein	0.0632
	TcSNP281	1	*Tc01v2_p034810* 60 S ribosomal protein L31	0.0226
	TcSNP1366	1	*Tc01v2_p025790* Heparan-alpha-glucosaminide N-acetyltransferase	0.0197
Wet bean mass	TcSNP1366	1	*Tc01v2_p025790* Heparan-alpha-glucosaminide N-acetyltransferase	0.0442
	TcSNP597	2	*Tc02v2_p004390* 21 kDa seed protein	0.038
	TcSNP281	1	*Tc01v2_p034810* 60 S ribosomal protein L31	0.0297
Seed number	TcSNP281	1	*Tc01v2_p034810* 60 S ribosomal protein L31	0.0638
	TcSNP1370	1	*Tc01v2_p008280* Histone H3.3	0.0611
	TcSNP597	2	*Tc02v2_p004390* 21 kDa seed protein	0.0483
Cotyledon length	TcSNP1102	1	*Tc01v2_p006960* Cytochrome c oxidase subunit 6a, mitochondrial	0.0545
	TcSNP1366	1	*Tc01v2_p025790* Heparan-alpha-glucosaminide N-acetyltransferase	0.0535
	TcSNP281	1	*Tc01v2_p034810* 60 S ribosomal protein L31	0.0475
Cotyledon mass	TcSNP139	8	*Tc08v2_p007790* Putative Cytochrome B5 isoform D	0.0221
	TcSNP17	2	*Tc02v2_p029690* RING/U-box superfamily protein, putative	0.0204
	TcSNP32	4	*Tc04v2_p022290* Pyruvate, phosphate dikinase, chloroplastic	0.0144
Fruit length	TcSNP1011	1	*Tc01v2_p028610* Aspartyl protease family protein 2	0.0386
	TcSNP1258	2	*Tc02v2_p004580* Probable aquaporin PIP2-8	0.0109
	TcSNP189	8	*Tc08v2_p000820* Succinate dehydrogenase subunit 5, mitochondrial	0.0108
Fruit surface anthocyanin	TcSNP174	4	*Tc04v2_p009180* 6-phosphogluconate dehydrogenase, decarboxylating 3	0.0244
	TcSNP401	4	*Tc04v2_p010060* ATP-dependent Clp protease proteolytic subunit 3, chloroplastic	0.0218
	TcSNP591	1	*Tc01v2_p009870* 60 S ribosomal protein L4-1	0.0199

### Genomic hotspots of trait association

To visualize the global distribution of the top-ranked marker–trait pairs and identify genomic regions associated with multiple traits, the top three SNPs for each of the 27 traits were plotted according to their physical position on the cacao chromosomes (Fig. S2). This approach revealed several genomic regions, or “hotspots,” that contained high-importance SNP–trait pairs for multiple, diverse traits. A notable pattern emerged where these hotspots were often concentrated near the boundaries of the chromosomes. This was particularly evident on chromosomes 1, 2, 5, 9, and 10, which all showed clear clusters of associations near their starting positions, while chromosomes 1, 2, 4, and 9 also showed clusters near their ends.

### Hierarchical clustering reveals functional trait groups

**Fig. 3:**
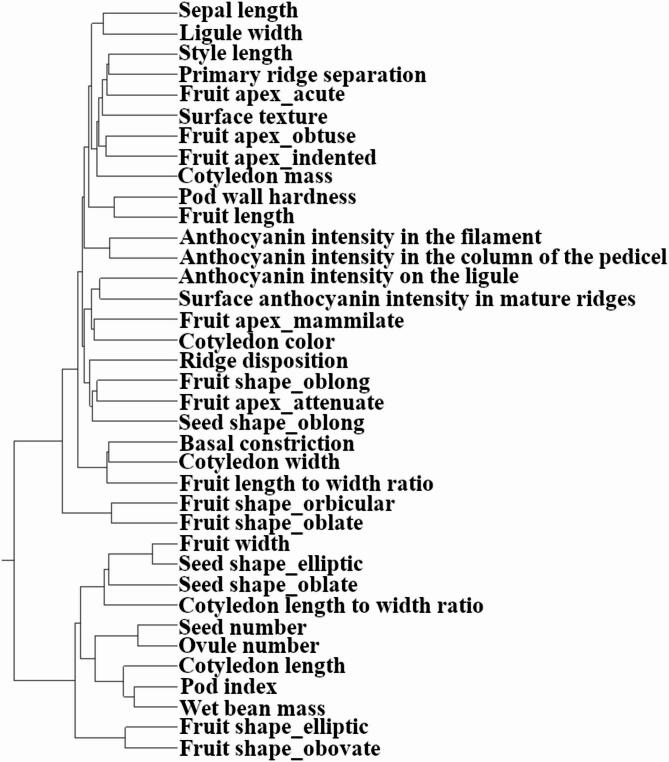
Hierarchical clustering of cacao traits based on corrected genetic association profiles. The dendrogram illustrates the relationships among traits, generated using Ward’s method on the SNP importance scores (Portion) from the corrected Bootstrap Forest GWAS. This analysis groups traits that share similar underlying genetic associations, revealing distinct functional clusters such as those related to overall yield and pigmentation.

To understand how selection might act on suites of related traits, we conducted hierarchical clustering on the ancestry-adjusted SNP importance scores derived from the Bootstrap Forest GWAS. The resulting dendrogram visualizes the relationships between traits based on their shared genetic architecture (Fig. 3).

A number of expected, functionally related traits were successfully grouped. For instance, the four measured anthocyanin intensity traits (in the filament, pedicel, ligule, and mature fruit ridges) were found in close proximity within the same parent cluster. Similarly, various fruit and seed shape traits formed logical, though not always adjacent, groupings.

The analysis revealed a distinct cluster composed of key yield-related traits. Key productivity metrics such as pod index and wet bean mass formed a tight pair, which in turn clustered with another group containing seed number, ovule number, and unexpectedly cotyledon length. Cotyledon width grouped with fruit characteristics and cotyledon length-to-width ratio grouped with seed shape traits and fruit width. Cotyledon mass was placed on a distant branch of the dendrogram, positioned in proximity to traits including pod wall hardness, fruit length, and several fruit apex form traits. The grouping of these distinct but related traits indicates a shared genetic network may govern overall yield potential in cacao.

### Comparison of gene ontology enrichment between naïve and structured models

To understand the biological functions of the genes associated with key traits, we performed a Gene Ontology (GO) enrichment analysis. We used the pod index as a case study to demonstrate the importance of correcting for population structure in a GWAS.

**Fig. 4:**
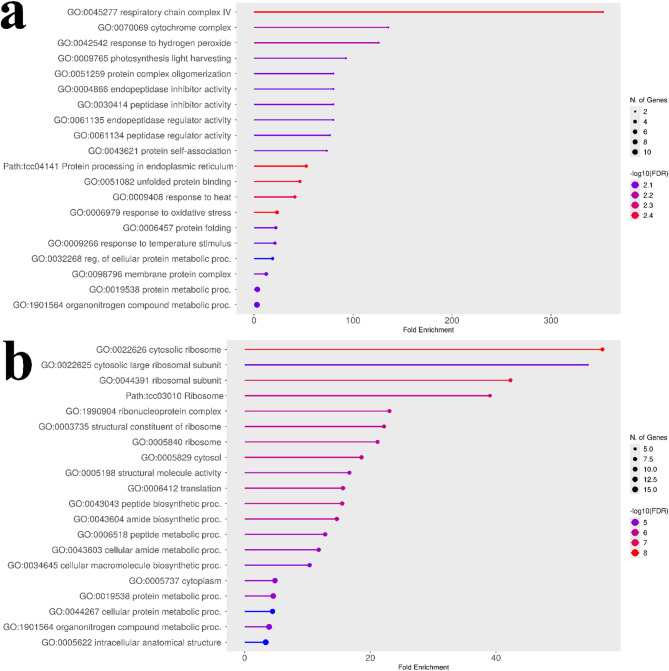
Comparison of GO enrichment analyses for uncorrected and corrected pod index GWAS results. The plots show enriched GO terms (FDR < 0.05) for the top candidate genes identified for the pod index trait. (**a**) GO enrichment plot for genes from the naïve (uncorrected) analysis. The analysis reveals a broad mix of pathways related to energy metabolism, protein processing, and stress response. Detailed KEGG pathways for protein processing in the endoplasmic reticulum and photosynthesis are shown in Figs. S3 and S4, respectively. (**b**) GO enrichment plot for genes from the structured association (corrected) analysis. After correcting for population structure, the result is a specific enrichment for the ribosome and protein synthesis pathways. A detailed KEGG pathway for the ribosome is shown in Fig. S5. This comparison illustrates how the statistical correction filtered out confounding signals to reveal the core machinery of protein synthesis as the primary biological process associated with pod index. Underlying GO enrichment tables (including FDR and −log10(FDR)) are provided in Supplementary Data S1 (sheets: Naïve GO Results, Corrected GO Results).

The GO analysis of the top candidate genes from the naïve (uncorrected) model revealed a broad mix of plausible biological pathways (Fig. 4a). The results were enriched for terms related to general energy metabolism (e.g., “respiratory chain complex,” “photosynthesis light harvesting”), protein management (e.g., “protein processing in endoplasmic reticulum”), and stress response (e.g., “response to hydrogen peroxide,” “response to heat”). This broader set of enriched terms may reflect ancestry-linked differences in baseline physiology in this structured panel; accordingly, we treat the naïve functional summaries as potentially confounded and use them only as a contrast to the structure-adjusted outputs. Detailed KEGG pathway diagrams^24–26^ for representative naïve enrichments are shown in Supplementary Figs. S3 and S4.

In contrast, the GO analysis of the top genes from the structured association (corrected) model tells a much more specific and strongly enriched pattern (Fig. 4b). After correcting for population structure, the broad metabolic and stress signals were reduced, and the result was strongly enriched for a single theme: the ribosome (FDR ≈ 10⁻⁸). Multiple enriched terms, such as “cytosolic ribosome,” “ribosomal subunit,” and “translation,” all point to the core machinery of protein synthesis. A detailed KEGG pathway diagram showing the specific ribosomal components identified is provided in Fig. S5. This direct comparison is consistent with the utility of the statistical correction, moving the analysis from a broad signal to a specific and robust biological insight that implicates a link between pod index and the plant’s capacity for protein production.

### Combined-trait analysis reveals core biological processes

**Fig. 5:**
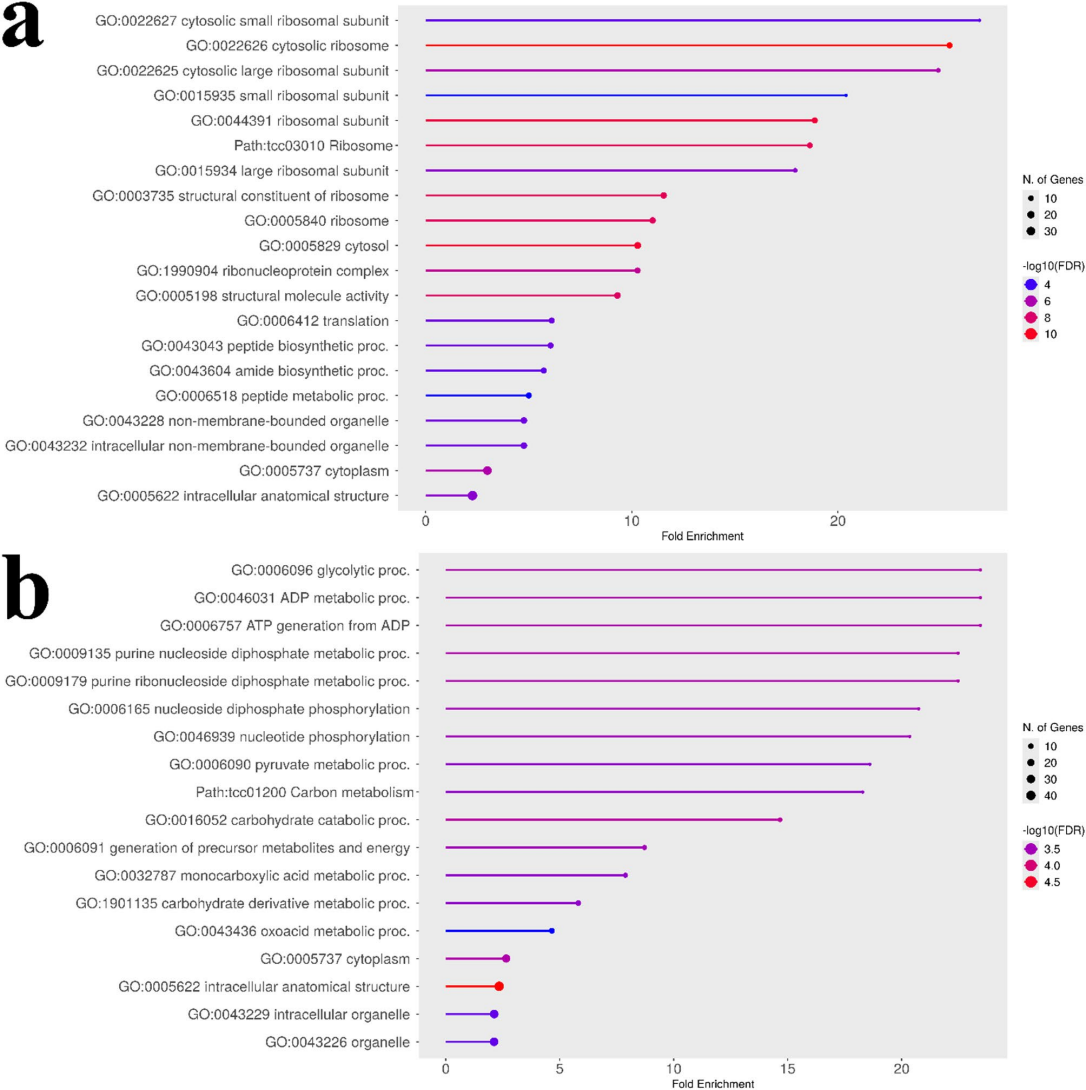
Comparison of GO enrichment plots for combined trait groups. (**a**) GO enrichment plot for the combined “yield & size” traits. The analysis is overwhelmingly dominated by terms related to the ribosome and protein synthesis. (**b**) GO enrichment plot for the combined “color & pigmentation” traits. This analysis reveals a strong enrichment for pathways related to primary energy metabolism. Underlying GO enrichment tables (including FDR and −log10(FDR)) are provided in Supplementary Data S1 (sheets: Naïve GO Results, Corrected GO Results).

To identify the fundamental pathways underpinning groups of related traits, we performed combined GO enrichment analyses on our corrected GWAS results. First, to identify the fundamental biological pathways underpinning this group of traits, we combined the candidate genes from nine size- and yield-related traits (ovule number, fruit length, fruit width, seed number, wet bean mass, cotyledon length, cotyledon width, cotyledon mass, and pod index). The GO enrichment analysis revealed a highly specific enrichment for the ribosome and protein synthesis (Fig. 5a). This result suggests that candidate summaries for the yield & size trait group are enriched for ribosome/protein-synthesis functions, consistent with a role for protein-production capacity in these traits.

To further validate our analytical process, we investigated the genetic basis of pigmentation across tissues. We performed a combined analysis of five color-related traits (flower anthocyanin intensity in the pedicel, ligule, and filament; fruit surface anthocyanin intensity; and seed cotyledon color). This analysis revealed a strong enrichment for pathways related to primary energy metabolism, including glycolysis and ATP generation (Fig. 5b), indicating that producing the anthocyanin pigments is an energetically expensive process. A key KEGG pathway identified in this analysis was “photosynthesis - antenna proteins,” and a detailed diagram showing the specific components identified, such as *Lhcb4*, is provided in Fig. S6.

**Fig. 6:**
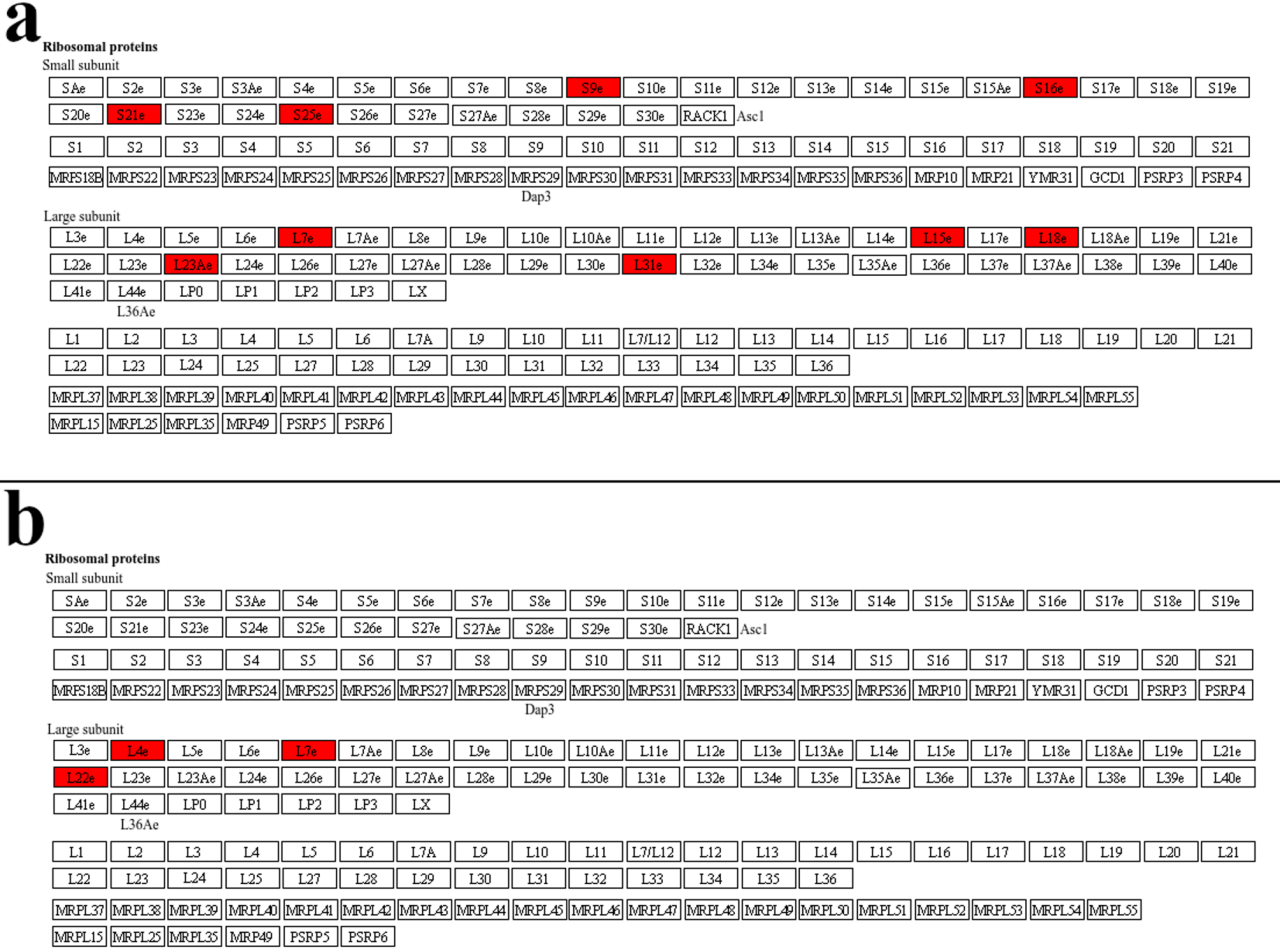
Comparison of ribosome KEGG pathway enrichment for the combined trait groups. Genes highlighted in red represent candidate genes identified from the ancestry-corrected GWAS. The figure displays a standardized KEGG reference map for the ribosome, which includes examples from different domains of life. (**a**) Ribosome pathway for the “yield & size” group. Numerous components of both the small (e.g., S9e, S16e) and large (e.g., L23Ae, L31e). (**b**) Ribosome pathway for the “color & pigmentation” group. A smaller, more specific set of ribosomal proteins (e.g., L4e, L7e, L22e) is highlighted, suggesting that pigmentation may be linked to specific regulatory functions of the ribosome. The KEGG pathway map file was obtained from the KEGG database (https://www.kegg.jp/kegg/kegg.html).

A deeper look at the top KEGG pathway results provided a more nuanced story (Fig. 6). For the “yield & size” group, numerous components of both the small and large ribosomal subunits were identified (Fig. 6a). This suggests that overall yield is linked to the general capacity and throughput of the entire protein factory. In contrast, for the “color & pigmentation” group, only a few, specific ribosomal proteins were identified, such as L4e, L7e, and L24e (Fig. 6b). This suggests that the genetic control of pigmentation may be linked to these specific ribosomal proteins having specialized regulatory roles, rather than affecting the overall rate of protein synthesis.

### Corrected GWAS links distinct morphological traits to specific biological pathways

**Fig. 7:**
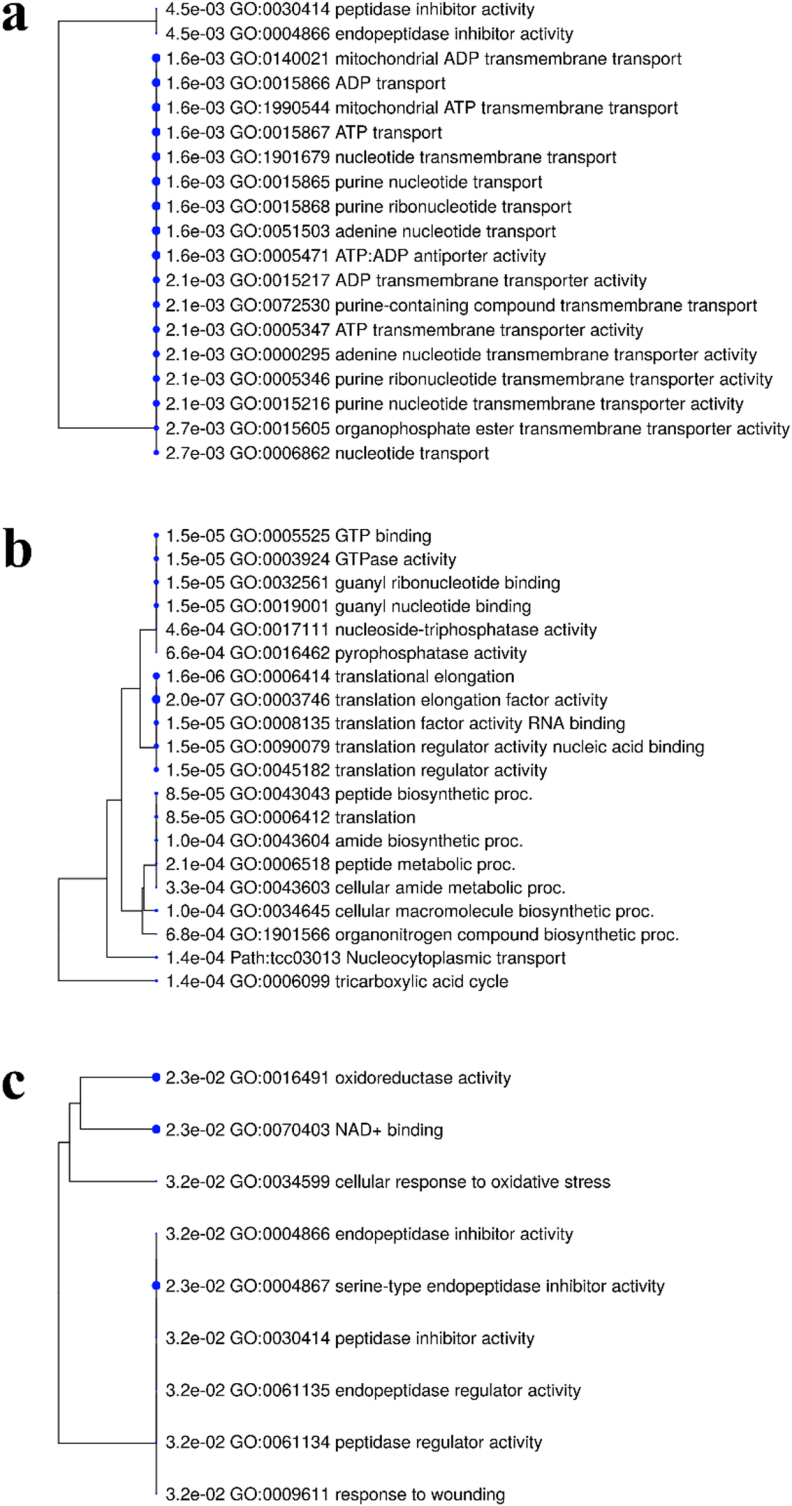
GO enrichment trees for notable individual morphological traits from the corrected GWAS. Each panel displays the top enriched GO terms for a specific trait. (**a**) GO enrichment for fruit shape - elliptic, dominated by pathways for mitochondrial ATP and nucleotide transmembrane transport. (**b**) GO enrichment for fruit husk hardness, showing a strong association with the TCA cycle and the machinery of translation and protein synthesis. (**c**) GO enrichment for fruit apex form - attenuate, highlighting pathways related to cellular stress response and the regulation of protein activity.

To demonstrate the breadth of biological mechanisms identified by our corrected GWAS, we highlighted the GO enrichment results for three distinct morphological traits (Fig. 7).

The analysis for fruit shape - elliptic revealed an overwhelming enrichment for pathways related to nucleotide and ATP transport across mitochondrial membranes (Fig. 7a). This specific finding suggests that the developmental program creating an elliptic shape is strongly linked to the efficient transport and delivery of cellular energy (ATP) and genetic building blocks (nucleotides) to support localized growth.

In contrast, the analysis for Fruit Husk Hardness pointed to the fundamental machinery of protein synthesis and cellular energy production (Fig. 7b). The top-ranked terms were related to the tricarboxylic acid (TCA) cycle, which is the central hub of cellular respiration, and numerous terms related to translation and peptide biosynthesis.

Finally, the analysis for fruit apex form (‘attenuate’, a shape where the fruit’s tip gradually tapers to a long, slender point) implicated pathways involved in stress response and regulation (Fig. 7c). Enriched terms included cellular response to oxidative stress, response to wounding, and various peptidase inhibitor activities. This suggests that the development of this specific shape is tied to the genetic pathways that manage cellular stress and regulate protein turnover.

### Predictive modeling of wet bean mass using Neural Networks

The pod index is calculated using the following formula:

$$\text{Pod Index}=\frac{1000}{\text{average dried cotyledon mass in grams}\times\text{average number of seeds per pod}}$$

**Fig. 8:**
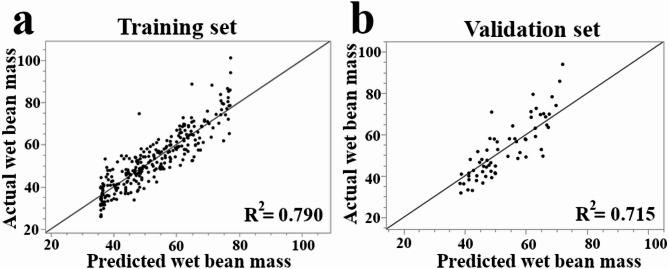
Evaluation of the Neural Boosted model for predicting wet bean mass in cacao, based on phenotypic data. The model was evaluated on the full dataset (*n* = 346) using a 5-fold cross-validation procedure. (**a**) Actual vs. predicted values aggregated from the training folds. (**b**) Actual vs. predicted values aggregated from the validation folds. The black line in each plot represents a perfect prediction (actual = predicted). The R-squared values summarize out-of-fold performance under 5-fold cross-validation; fold-wise validation *R*² distributions across candidate models are shown in Fig. 9.

**Fig. 9:**
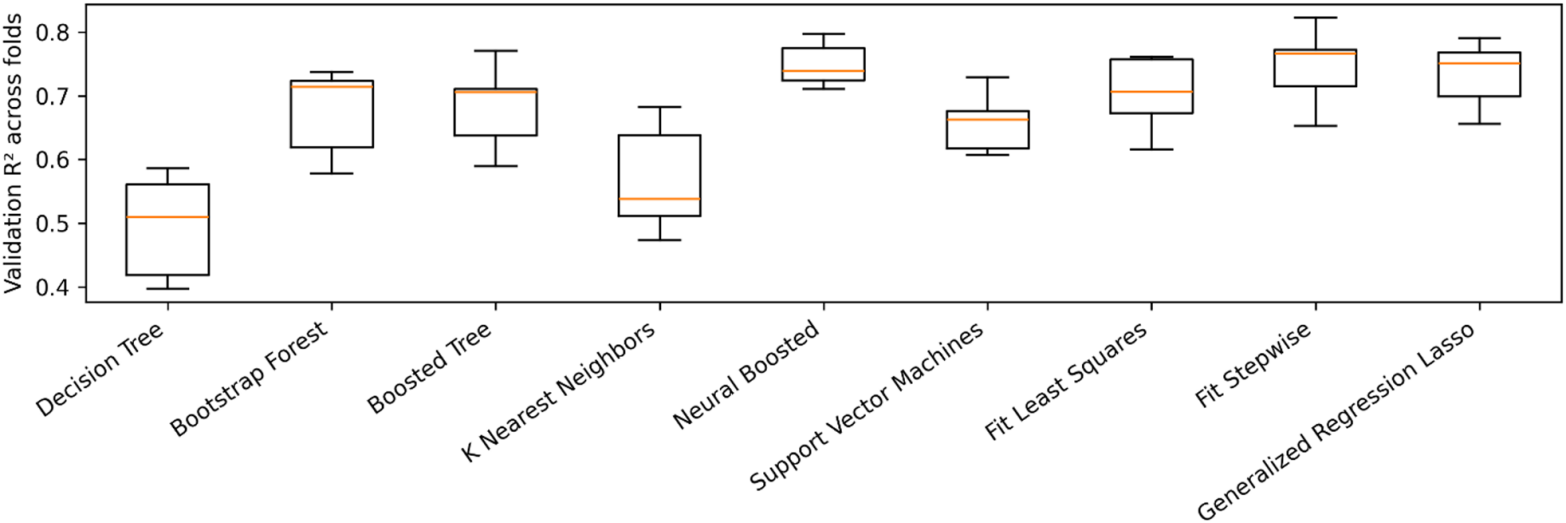
Fold-wise cross-validation performance across candidate prediction models for wet bean mass. Boxplots show the distribution of validation *R*² across the five folds of 5-fold cross-validation for each method evaluated during model screening. Reporting fold-wise distributions (rather than a single value) reflects variability across data splits and supports comparison of predictive performance across methods.

This calculation determines the number of pods required to produce one kilogram (1000 g) of dried cocoa beans. A lower pod index value indicates a higher yield potential, as fewer pods are needed to produce the same amount of cocoa. Wet bean mass provides a more direct assessment of yield, so we employed a machine learning approach using Neural Networks to identify predictive phenotypic traits while excluding obvious correlating traits such as seed number, fruit length, fruit width, and fruit length to width ratio. The performance of the trained Neural Boosted model is shown in Fig. 8. Model performance was evaluated by 5-fold cross-validation, and fold-wise validation *R*² is reported as mean ± SD across folds (Fig. 9). The scatter plots of actual vs. predicted wet bean mass values show a close alignment of the points with the diagonal line representing perfect prediction, indicating agreement between observed and out-of-fold predicted wet bean mass values under 5-fold cross-validation. Further analysis using the Neural Boosted model identified cotyledon mass as the primary driver of wet bean mass, with a remarkably high importance score of 6 (Table S1). Cotyledon length emerged as the second most influential predictor, receiving an importance score of 2. Cotyledon length-to-width ratio showed slight predictive power with an importance score of 1, whereas cotyledon width and other phenotypic traits showed little to no predictive influence (Table S1). Cotyledon mass displayed a substantial positive influence on wet bean mass, evidenced by its main effect of 0.484 and total effect of 0.5198. This suggests that cotyledon mass impacts wet bean mass both directly and indirectly through interactions with other variables. Cotyledon mass (portion = 0.520) and cotyledon length (portion = 0.213) accounted for the vast majority (73.3%) of the predictive power in the model, highlighting their dominant roles in determining wet bean mass.

## Discussion

### Correlation between patristic distances and phenotypic traits

We first examined how ancestry relates to phenotypic variation in this cacao panel (*n* = 342 accessions with both genotype and phenotype records for the Mantel analyses). The results indicate that broad ancestry accounts for a limited fraction of phenotypic divergence in this panel: significant traits show small positive patristic–phenotypic correlations, with the strongest for pod index (*r* = 0.25, *q* = 0.0019), followed by cotyledon width (*r* = 0.20, *q* = 0.0019). While genetic divergence has increased trait difference for these two traits, the weak overall correlation between patristic distances and phenotypic traits suggests that broad phylogenetic relationships are not the primary determinants of cacao phenotypic variation in this collection.

### Population-structure correction sharpens candidate prioritization and functional summaries for yield components

We use fixed vs. unfixed analyses as a panel-specific sensitivity comparison to illustrate how population structure can alter SNP-importance rankings and downstream functional summaries. The naïve analysis highlighted a top SNP in a peroxidase gene, a plausible association that may also be sensitive to ancestry-linked confounding in structured panels^27–29^ that could lead to spurious associations if population structure is not controlled^30,31^. In contrast, the corrected model elevated TcSNP597 to the top-ranked position, a locus within a gene for a 21 kDa proteinase inhibitor seed protein, a class of proteins known to be major determinants of seed quality and nutritional value^32^. This shift is notable, as it moves the focus from a general stress-response gene to a gene class with a specific role in seed development and defense, making it a more direct and plausible candidate. Similarly, the correction refined the associated pathway: the uncorrected results were enriched for the broad but relevant pathway of “protein processing in the endoplasmic reticulum,” while the corrected results pinpointed the core of this process, with ribosome/translation-related terms becoming more prominent in the adjusted candidate set. These comparisons illustrate that structure adjustment can yield more focused, hypothesis-generating functional summaries in this dataset.

### Recurrent candidates across yield components are consistent with partially shared genetic influences

Several loci recur among top-ranked candidates across correlated yield components (Table 1), consistent with partially shared genetic influences (pleiotropy) in complex traits. Given marker density and phenotype provenance limitations, we treat these loci as candidates for follow-up rather than evidence of a single controlling locus. Pleiotropy is a key feature of complex traits in major crops, where single loci often influence multiple, correlated yield components^33,34^. Specifically, TcSNP281, TcSNP597, and TcSNP1366 were identified as top associations for combinations of pod index, wet bean mass, and seed number (Table 1). The putative genes at these loci are involved in fundamental biological processes: protein synthesis (60 S ribosomal protein L31), seed development and defense (21 kDa proteinase inhibitor seed protein, known as a negative regulator for seed quality), and energy metabolism (heparan-alpha-glucosaminide N-acetyltransferase). The recurrence of these loci across yield components is consistent with partially shared genetic influences in this dataset.

The validity of our analytical approach is demonstrated by an internal positive control. The analysis for fruit surface anthocyanin recovered 6-phosphogluconate dehydrogenase (TcSNP174), a key enzyme biochemically upstream of flavonoid precursors. This finding, combined with the identification of a locus (TcSNP401) that converges with an independently reported finding for cacao pigmentation, provides a biologically coherent check that our structured model successfully identifies pathway-specific, non-vigor associations. 6-phosphogluconate dehydrogenase catalyzes a rate-limiting step in the oxidative pentose phosphate pathway (OPP)^35^. The OPP pathway is critical as it generates carbon precursors for the shikimate pathway, a direct link that has recently been genetically demonstrated through the activity of 6-phosphogluconate dehydrogenase^36^. The shikimate pathway, in turn, is the well-established route for the biosynthesis of aromatic amino acids and subsequently the entire class of flavonoids, including the anthocyanin pigments that determine color^37,38^. This pigmentation example provides a biologically coherent check: a top-ranked marker maps to a gene in the oxidative pentose phosphate pathway, which is upstream of precursor supply for flavonoid/anthocyanin biosynthesis; this supports pathway-consistent candidate recovery without implying causality.

### From predictive models to biological mechanisms

Our findings can be contextualized by comparing them to the initial analysis of this germplasm by Bekele et al.^11^. It is noteworthy that both studies, despite using different corrected GWAS models (Bootstrap Forest vs. MLM), independently identified TcSNP401 as a key locus associated with fruit surface anthocyanin. This convergence across methods applied to the same underlying panel supports the relevance of this region for pigmentation and provides a useful internal consistency check. Beyond this convergence, our machine learning-based approach identified a largely distinct set of top candidate genes for most traits, suggesting our analysis has captured different facets of the genetic architecture and provides a complementary view of cacao yield genetics.

Furthermore, this study successfully created a bridge between predictive modeling and genetic markers. Our Neural Networks independently identified cotyledon mass and length as the most powerful predictors of final wet bean mass. Our GWAS then provided a genetic basis for these predictive traits. Cotyledon length was among the top-ranked candidates with a SNP annotated to a mitochondrial cytochrome c oxidase subunit, consistent with prior evidence that cytochrome c oxidase activity is important for plant embryogenesis and seed viability^39,40^. Cotyledon mass was associated with a SNP in a RING/U-box superfamily protein. This finding is well-supported by studies in other crops showing that this class of E3 ubiquitin ligases directly regulates seed and organ size by targeting key proteins for degradation^41,42^.

Our combined-trait GO analysis revealed the fundamental processes underpinning major phenotypic groups. By combining all nine yield- and size-related traits, the analysis converged on a single, important biological theme: the ribosome and protein synthesis. A deeper look revealed that numerous components of both the small and large ribosomal subunits were associated with these traits, suggesting that overall yield potential is linked to the general capacity and throughput of the entire protein synthesis machinery. This conclusion is well-supported by studies showing that the disruption of individual ribosomal protein genes leads to significant defects in plant growth, development, and seed size^43–45^. In parallel, the combined analysis of five color- and pigmentation-related traits pointed directly to primary energy metabolism, including glycolysis and ATP generation. A key pathway identified in this group was “photosynthesis - antenna proteins,” highlighting the specific gene *Lhcb4*. Antenna proteins regulate how much light energy reaches photosystem II and variations in these proteins can directly impact chloroplast redox balance which is a primary trigger for anthocyanins as photoprotective agents. This provides a direct mechanistic link between the machinery of light capture and the energetically expensive process of pigment production^46,47^.

Beyond these broad themes, the analysis of individual corrected traits revealed a diversity of specific biological mechanisms controlling morphology. For instance, the genetics of fruit shape - elliptic were not linked to general growth but to the specific machinery of ATP and nucleotide transport across mitochondrial membranes. This suggests a key role for “energy logistics” in developmental patterning, a concept supported by findings that disrupting mitochondrial transporters can severely hamper organ development and morphology^48,49^. In another example, fruit husk hardness was strongly associated with the Citrate (TCA) cycle and the machinery of translation. This provides a direct link between a physical property and the cell’s core metabolic and biosynthetic capacity, as studies have shown that restricting TCA cycle activity directly impairs the synthesis of secondary cell wall components like cellulose and lignin that confer structural rigidity^50,51^. These findings showcase the complexity of developmental genetics and demonstrate our approach’s ability to uncover novel and highly specific hypotheses for a wide range of traits.

Hierarchical clustering of traits based on the ancestry-adjusted GWAS results provided a global view of the genetic architecture, revealing several functionally important groupings (Fig. 3). The analysis partitioned the traits into distinct clusters, most notably grouping key productivity metrics such as pod index, wet bean mass, seed number, and cotyledon length. This supports the concept of a shared genetic foundation for overall yield and corroborates the pleiotropic effects suggested by the single-trait GWAS, where the same SNPs were associated with multiple of these traits. Clustering of trait–gene association profiles underscored the modular nature of yield components. Key productivity metrics, pod index and wet bean mass, formed a tight pair and clustered with seed number (and ovule number, cotyledon length), consistent with a shared genetic backbone for overall yield. By contrast, cotyledon mass lay on a distant branch of the dendrogram, proximate to pod wall hardness, fruit length, and several apex‑form traits. This separation indicates that, although phenotypically related, the genetic factors governing individual bean mass are partly distinct from those shaping bean count and pod‑level characteristics. Practically, this suggests that bean size and bean number can be improved as semi‑independent targets in selection schemes, rather than assuming one will automatically track the other.

Beyond the clustering of traits, our global analysis of SNP locations revealed a striking pattern in the underlying genetic architecture. By mapping the top three associated SNPs for all 27 traits, we identified several genomic “hotspots” containing associations for multiple, diverse traits (Fig. S2), a phenomenon that has been observed for important agronomic traits in other major crops^52,53^. A distinct trend emerged where many of these hotspots were concentrated in the subtelomeric regions of the chromosomes, particularly on Chromosomes 1, 2, 4 and 9. These subtelomeric regions are known to be dynamic parts of the genome, often characterized as being gene-rich and exhibiting high rates of recombination^54,55^. These observations have motivated hypotheses that subtelomeric regions can be evolutionarily dynamic; however, in our low-density panel we treat the apparent enrichment of top-ranked SNPs near chromosome ends as descriptive and hypothesis-generating^56,57^. This concentration of trait-associated loci suggests these dynamic genomic regions may have been key targets of selection during both the initial domestication of cacao and its subsequent, geographically diverse improvement.

### Implications for cacao breeding and future directions

The successful prediction of wet bean mass using Neural Networks (Fig. 8), well-suited for capturing complex, non-linear relationships often found in biological systems, demonstrates the potential of leveraging phenotypic data for developing predictive tools for complex traits in cacao^58^. The model’s high accuracy (R-squared = 0.790 for training, 0.715 for validation) highlights the strong relationships between the predictor variables and wet bean mass. Notably, cotyledon mass and cotyledon length were identified as the two most important predictors (Table S1) of wet bean mass, a direct measure of yield. Larger cotyledons on young cacao seedlings may be indicators of vigor and higher photosynthetic capacity, which over the long-term could result in more productive cacao trees, a principle supported in other crops where early-stage growth traits have been shown to be predictive of final yield^59^. While this suggests that simple measurement of these two traits could provide an accurate and cost-effective method for predicting productivity, further longitudinal evidence is required to demonstrate early screening utility of these key-traits. Nonetheless, the identification of these early-screening traits may have important implications for cacao breeding programs that are often hampered by long generation times and an urgent need for new, high-yielding varieties^13^. Targeting specific combinations of traits, guided by the identified clusters (Fig. 3) and the machine learning model (Fig. 8), could lead to even more efficient selection strategies for improving yield potential. Furthermore, these results, combined with our GWAS findings, suggest that genomic selection, incorporating information from multiple loci across the genome, could be a powerful tool for accelerating cacao improvement, an approach particularly impactful for perennial crops with long breeding cycles^60^.

The findings of this study, particularly the successful application of Neural Networks for predicting wet bean mass, provide a springboard for several promising avenues of future research in cacao. Given the potential impact on breeding strategies, prioritizing the validation of candidate genes identified through GWAS would be beneficial. This could involve gene expression studies and functional analyses using transgenic approaches or, more directly, CRISPR-Cas9-mediated gene editing, which is a critical step for validating the causal effect of candidate genes identified through association studies^61^. Second, expanding the association mapping and genomic selection studies to larger and more diverse populations of cacao, including different genetic groups and geographic origins, would help to confirm the identified associations and identify additional loci contributing to trait variation across a wider range of germplasm. This could also involve exploring genotype-by-environment interactions by evaluating the performance of diverse accessions in multiple locations in collaboration with international partners. Third, investigating the role of other types of genetic variants, such as copy number variations and structural variants, could improve predictive power. Fourth, integrating genomic data with other omics data, such as transcriptomic, proteomic, and metabolomic profiles, could further elucidate the molecular mechanisms underlying trait variation. Both the inclusion of structural variants and the use of multi-omics data are considered key future directions for enhancing genomic prediction models^60^. Moreover, exploring different Neural Network architectures, such as the deep learning methods that are increasingly being harnessed for genomic prediction, could provide even greater accuracy when modeling complex traits from high-dimensional data^62^.

This study provides a comprehensive analysis of the phenotypic and genotypic variation in a diverse cacao collection, dissecting the complex relationships between population structure, genetic markers, and key agronomic traits. A central outcome was the demonstration of the profound impact of population structure on association mapping. By applying an ancestry-adjusted model, our GWAS successfully filtered out misleading signals related to general plant ‘vigor’ to instead highlight ribosome/translation-related terms among yield-component candidate summaries after structure adjustment, treated as hypothesis-generating signals given marker density and phenotype provenance constraints. This corrected approach led to the identification of a small set of loci which were responsible for influencing multiple yield components like pod index, wet bean mass, and seed number. The hierarchical clustering of these corrected results further illuminated the genetic landscape, partitioning traits into distinct functional groups related to yield and pigmentation. Moreover, a highly accurate machine-learning model identified cotyledon mass and length as the most powerful predictors of wet bean mass, a direct measure of yield. While this work provides a phenotype-only prediction example; because top predictors are closely related to the response trait, breeding utility as an early-stage proxy requires longitudinal validation. Collectively, these findings enhance our understanding of the genetic architecture of yield in cacao and provide a powerful, framework for developing superior varieties through modern breeding strategies.

## Materials and methods

### Phenotype and genotype data

Phenotypic and genotypic data used in this study were obtained from a publicly available, large-scale assessment of cacao germplasm detailed in Bekele et al.^11^. The source study evaluated 421 cacao accessions from the International Cocoa Genebank, Trinidad (ICGT); all analyses in the present study were restricted to the subset with available SNP genotypes after filtering and phenotype values for the focal traits (up to *n* = 346 accessions)^11,63^.

Phenotypic evaluations for 27 underlying traits, encompassing flower, fruit, and seed characteristics, were derived from the dataset by Bekele et al.^11^. Several categorical traits (e.g., fruit/seed shape and apex form) were encoded as binary indicator variables for modeling; therefore, the modeling matrix contains more columns than 27, while still representing the same 27 underlying traits. Flower traits included anthocyanin intensity in the column of the pedicel, sepal length, anthocyanin intensity on the ligule, ligule width, anthocyanin intensity in the filament, style length, and ovule number. Fruit traits included shape, basal constriction, apex form, surface texture, surface anthocyanin intensity in mature ridges, ridge disposition, primary ridge separation, pod wall hardness, fruit length, fruit width, and fruit length to width ratio. Seed traits included number, shape, cotyledon color, total wet bean mass, cotyledon length, cotyledon width, cotyledon mass, and cotyledon length to width ratio. Pod index, a key agronomic trait that reflects yield potential in cacao, was also included as a composite trait^11^.

Phenotype availability varies by trait in the public dataset; analyses were performed on trait-specific subsets with non-missing values. The full accession inclusion and trait-wise availability are provided in Supplementary Data S1. For the genotypic data, Bekele et al.^11^ targeted a total of 836 SNPs located in coding sequences, selected based on their similarity to known protein sequences. After filtering, 671 SNPs were retained for downstream analyses. Consequently, the dataset analyzed here consists of 671 gene-centric SNPs and phenotype data for up to 346 accessions (trait-specific missingness as above). Because marker coverage is sparse and non-uniform, association results are interpreted as candidate-prioritization signals rather than fine-mapping of causal loci. Because the retained SNPs are gene-centric (coding-region) markers, the panel is enriched for annotated genes and is therefore suitable for candidate prioritization and functional theme summaries, while remaining underpowered for genome-wide fine-mapping.

### Correlation between patristic distances and phenotypic traits

We tested whether more distantly related accessions are more phenotypically different by performing Mantel tests (Pearson, 9,999 permutations) between the patristic distance matrix and pairwise phenotypic distances for each trait. Patristic distances were computed in Geneious (v2025.2.1). Accessions lacking overlap between the phylogeny and the genotype/phenotype records were excluded, yielding a final intersection set (n reported in Results for the Mantel analyses).

### Analysis of population structure and phenotypic variation using PCA

To visualize the underlying structure of the phenotypic and genotypic variation among the cacao accessions, and to generate covariates for population structure correction, PCA was performed using JMP Pro 17. Prior to analysis, the categorical SNP data was processed. First, missing SNP genotypes (coded as ‘N’) were imputed using the Multivariate SVD Imputation method within the ‘Explore Missing Values’ platform of JMP Pro 17, using 6 singular vectors and a maximum of 10 iterations. Following imputation, the complete categorical SNP matrix was converted into a numerical format based on allele counts (e.g., 0, 1, 2) using JMP Pro’s built-in functionalities to serve as input for the analysis. Two separate PCAs were then conducted. The first was performed on the matrix of all measured phenotypic traits to visualize phenotypic relationships. The second was performed on the final imputed and numerically-coded SNP genotype matrix to assess population structure. Principal Component Scores for the first three components were saved back to the main data; PC1–PC3 explained 16.0%, 11.8%, and 9.4% of the SNP variance, respectively. To create the corrected phenotype for the GWAS, these three PCs were then used as predictors in a standard least squares linear regression model for each trait, and the resulting residuals were saved as the new, ancestry-corrected target variable.

### Machine learning-based GWAS to associate markers with traits

To identify genomic regions associated with phenotypic variation in the cacao accessions, we employed a machine learning GWAS approach based on Bootstrap Forest models implemented in JMP Pro 17. Unlike traditional linear models, which primarily test for additive effects of single markers, ensemble ML methods like Bootstrap Forest can model non-linear relationships among predictors; in this study, we use these models for candidate prioritization rather than explicit interaction mapping.

To create a comprehensive view of the genetic architecture, two parallel analyses were conducted: a naïve model using raw phenotypes and a structure-adjusted model using ancestry-adjusted residual phenotypes. Specifically, for each trait we fit a linear model with PC1–PC3 as predictors and saved residuals as ancestry-adjusted targets; Bootstrap Forest models were then trained on (i) raw phenotypes (naïve) and (ii) residual phenotypes (structure-adjusted) using SNP markers as predictors.

The naïve model based analysis provides an exploratory view of the entire genetic landscape, identifying all associations including broad effects potentially confounded with population structure. In contrast, the structured association model based analysis statistically removes the ancestral background noise to prioritize loci whose SNP-importance rankings are less sensitive to ancestry-linked structure in this panel. Comparing these two outputs provides a panel-specific sensitivity comparison of how population structure affects SNP-importance rankings and downstream candidate summaries; we avoid causal interpretation given marker density and phenotype provenance constraints.

The GWAS was conducted for all phenotypic traits, including those related to accession group, flower morphology, fruit morphology, seed characteristics, cotyledon and bean mass, and pod index. Pod index is a key agronomic trait that reflects yield potential, calculated as 1000/(average dried cotyledon mass × average number of seeds per pod)^11^. Importantly, it is a composite trait that takes into account both pod size and seed number, with lower pod index values indicating higher yield potential^11^. For the naïve model based analysis, separate Bootstrap Forest models were trained to predict each raw phenotypic trait using the full set of SNP markers as predictors.

For the structured association model based, we first accounted for confounding due to population structure using a two-step method. A linear model was used to predict each trait using the first three principal components (PC1, PC2, and PC3) from the genotypic PCA as predictors. The model can be represented by the formula:

*Y* = *β*₀ + *β*₁(PC1) + *β*₂(PC2) + *β*₃(PC3) + *ε*.

where Y is the vector of phenotypic values for a given trait, *β*₀ is the overall mean, PC1, PC2, and PC3 are the vectors of the principal component scores, *β*₁-*β*₃ are their corresponding fixed-effect regression coefficients, and ε is the vector of residuals.

The residuals from this model (*ε*), which represent the phenotypic variation independent of ancestry, were saved. Subsequently, separate Bootstrap Forest models were trained to predict these ancestry-corrected residuals for each trait using the full set of SNP markers as predictors.

For all Bootstrap Forest models in both analyses, default settings included the following parameters: number of trees in the forest was set to 100, the number of terms sampled per split was 1, the bootstrap sample rate was 1, the minimum number of splits per tree was 10, the maximum number of splits per tree was 2,000, and the minimum size split was 5. The top-ranking markers from both the corrected and uncorrected analyses were identified based on their importance scores, calculated as the “Portion” in JMP Pro. Given the sparse marker density, we did not treat individual SNPs as resolving causal variants. Candidate genes were assigned conservatively based on the annotated gene containing the SNP (coding sequence) or the nearest annotated gene in the Criollo v2 reference annotation (GCF_000208745.1).

### Gene ontology enrichment analysis

A GO enrichment analysis was conducted to investigate the potential biological functions and pathways overrepresented among the candidate genes associated with key agronomic traits. To compare the effects of population structure correction, this analysis was performed on gene lists generated from both the naïve (uncorrected) and structured association (corrected) models for a subset of important traits, including pod index, wet bean mass, and anthocyanin intensity on the ligule.

For each analysis, a list of unique gene identifiers was compiled from all markers exceeding an importance cutoff (Portion > 0.005), used here as a pragmatic reporting threshold to define manageable candidate lists for downstream functional summarization (not a statistical significance threshold). Empirical calibration of this threshold across traits is provided in Supplementary Data S1. In cases where a marker could not be mapped to the reference genome, it was omitted from this downstream analysis. The curated list of unique *Theobroma cacao* gene IDs was submitted to ShinyGO v0.82^64^ (available at http://bioinformatics.sdstate.edu/go/*).* The enrichment analysis was conducted using the *Theobroma cacao* Belizean Criollo B97-61 B2 genome database. Default settings were applied, including a FDR cutoff of 0.05 to determine statistical significance. Pathway size filters were set to a minimum of 2 and a maximum of 5,000 genes per GO term.

### Hierarchical clustering of trait association profiles

To visualize the global genetic relationships between traits, we performed hierarchical clustering analysis. This analysis used the Ward method based on the importance scores (portion) derived from the structured Bootstrap Forest models for all traits. This approach groups traits that exhibit similar genetic association profiles, suggesting shared underlying genetic pathways or pleiotropic effects.

### Development of a machine learning model for predicting wet bean mass

To further investigate the complex relationships between the various measured traits and wet bean mass, a machine learning approach employing Neural Networks was utilized^65^. The primary goal was to train a predictive model for wet bean mass, a key agronomic trait in cacao, based on the suite of phenotypic characteristics used in this study. Model selection and training were performed using JMP Pro 17. An initial model screening step was conducted using the built-in model screening function to evaluate the performance of various algorithms and identify the most promising model type for predicting wet bean mass. The Neural Boosted model emerged as the best-performing model and was therefore selected for further analysis. The model was configured with a single hidden layer containing three neurons with a hyperbolic tangent (TanH) activation function (NTanH(3)) and built using 20 boosting iterations (NBoost(20)).

To assess predictive performance, a 5-fold cross-validation scheme was implemented on the full dataset (*n* = 346). Fold-wise validation performance is reported (mean ± SD across folds) to reflect variability across data splits (Fig. 9). The Neural Boosted model, with the specified NTanH(3) and NBoost(20) settings, was trained and validated under 5-fold cross-validation. Model performance was summarized as mean ± SD of validation *R*² across the five folds (Fig. 9). To reduce trivial leakage, we excluded a predefined set of closely related traits (seed number and fruit-size measures) from the predictor set; retained predictors and their importance scores are reported in Table S1. The performance of the trained model was assessed using the coefficient of determination (R-squared), summarized as mean ± SD of validation *R*² across the five folds. The importance of individual features (traits) in the Neural Boosted model was assessed to gain insights into the factors most strongly influencing wet bean mass prediction.

## Supplementary Information

Below is the link to the electronic supplementary material.

Supplementary Material 1

Supplementary Material 2

## Data Availability

The present study is a re-analysis of previously published, publicly available datasets. No new raw genotype or phenotype data were generated in this study. The raw phenotypic and genotypic data of the 346 Theobroma cacao accessions analyzed in this work are available in the Supporting Information of Bekele et al. (2022) at https://doi.org/10.1371/journal.pone.0260907. All derived results generated during the current study, including trait-importance rankings and GO enrichment results, are included in this published article (and its Supplementary Information files).

## References

[1] Díaz-Montenegro, J. Livelihood strategies and risk behavior of cacao producers in Ecuador: effects of national policies to support cacao farmers and specialty cacao landraces. PhD thesis, Universitat Politècnica de Catalunya (2019).

[2] Hall, J. N. Applying a One Health approach to study the livelihoods of cocoa farming communities in Bougainville. (2022).

[3] Bekele, F. & Phillips-Mora, W. Cacao (Theobroma cacao L.) breeding. *Adv. Plant. Breed. Strategies: Industrial Food Crops: Volume*. **6**, 409–487 (2019).

[4] Alden, D. The significance of cacao production in the Amazon region during the late colonial period: an essay in comparative economic history. *Proc. Am. Philos. Soc.* **120**, 103–135 (1976).

[5] Gardea, A. A. et al. Cacao (Theobroma cacao L.). *Fruit and Vegetable Phytochemicals: Chemistry and Human Health, 2nd Edition* 921–940 (2017).

[6] Kongor, J. E., Owusu, M. & Oduro-Yeboah, C. Cocoa production in the 2020s: challenges and solutions. *CABI Agric. Bioscience*. **5**, 102 (2024).

[7] Walters, D. *Chocolate Crisis: Climate Change and Other Threats to the Future of Cacao* (University Press of Florida, 2020).

[8] Boza, E. J. et al. Genetic characterization of the cacao cultivar CCN 51: its impact and significance on global cacao improvement and production. *J. Am. Soc. Hortic. Sci.* **139**, 219–229 (2014).

[9] Mustiga, G. M. et al. Phenotypic description of Theobroma cacao L. for yield and vigor traits from 34 hybrid families in Costa Rica based on the genetic basis of the parental population. *Front. Plant Sci.* **9**, 808 (2018). 29971076 10.3389/fpls.2018.00808 PMC6018478

[10] Izzah, N. K. et al. Improvement of Cacao Pod Characteristics and its Molecular Characterization in 4 F1 Cacao Populations. Preprint at Research Square 10.21203/rs.3.rs-4766155/v1 (2024).

[11] Bekele, F. L. et al. Genome-wide association studies and genomic selection assays made in a large sample of cacao (Theobroma cacao L.) germplasm reveal significant marker-trait associations and good predictive value for improving yield potential. *Plos one*. **17**, e0260907 (2022). 36201531 10.1371/journal.pone.0260907 PMC9536643

[12] Bediako, K. A., Padi, F. K., Obeng-Bio, E. & Ofori, A. Genetic diversity and parentage of cacao (*Theobroma cacao *L.) populations from Ghana using single nucleotide polymorphism (SNP) markers. * Plant Genet. Resour.* 23, 40–47 (2025).

[13] Gutiérrez, O. A., Campbell, A. S. & Phillips-Mora, W. Breeding for disease resistance in cacao. In Cacao Diseases: A History of Old Enemies and New Encounters (eds Bailey, B. & Meinhardt, L.) 567–609 (Springer, 2016).

[14] Rodriguez-Medina, C. et al. Cacao breeding in Colombia, past, present and future. *Breed. Sci.* **69**, 373–382 (2019). 31598069 10.1270/jsbbs.19011 PMC6776146

[15] Wickramasuriya, A. M. & Dunwell, J. M. Cacao biotechnology: current status and future prospects. *Plant Biotechnol. J.* **16**, 4–17 (2018). 28985014 10.1111/pbi.12848 PMC5785363

[16] McElroy, M. S. et al. Prediction of cacao (Theobroma cacao) resistance to Moniliophthora spp. diseases via genome-wide association analysis and genomic selection. *Front. Plant Sci.* **9**, 343 (2018). 29662497 10.3389/fpls.2018.00343 PMC5890178

[17] Romero Navarro, J. A. et al. Application of genome wide association and genomic prediction for improvement of cacao productivity and resistance to black and frosty pod diseases. *Front. Plant Sci.* **8**, 1905 (2017). 29184558 10.3389/fpls.2017.01905 PMC5694496

[18] Fernandes, L. S., Correa, F. M., Ingram, K. T., de Almeida, A. A. F. & Royaert, S. QTL mapping and identification of SNP-haplotypes affecting yield components of *Theobroma cacao* L. Hortic. **7**, 26 (2020). 10.1038/s41438-020-0250-3 PMC7049306 32140235

[19] Duarte-Carvajalino, J. M., Paramo-Alvarez, M., Ramos-Calderón, P. F. & González-Orozco, C. E. Estimation of canopy attributes of wild cacao trees using digital cover photography and machine learning algorithms. *iForest-Biogeosciences Forestry*. **14**, 517 (2021).

[20] Omas-as, A. M. & DAANG, J. A. M. ARBOLEDA, E. R. Machine Learning as a Strategic Tool: A Comprehensive Literature Review for Advancing Agricultural Analysis, with Emphasis on the Cocoa Bean Quality Assessment. *Int. J. Sci. Res. Eng. Dev.* **7**, 269 (2024).

[21] Tan, J., Balasubramanian, B., Sukha, D., Ramkissoon, S. & Umaharan, P. Sensing fermentation degree of cocoa (Theobroma cacao L.) beans by machine learning classification models based electronic nose system. *J. Food Process Eng.* **42**, e13175 (2019).

[22] Lamos-Díaz, H., Puentes-Garzón, D. E. & Zarate-Caicedo, D. -A. Comparison between machine learning models for yield forecast in cocoa crops in Santander, Colombia. Rev. Fac. Ing. **29**, 18 (2020).

[23] Breiman, L. Random forests. *Mach. Learn.* **45**, 5–32 (2001).

[24] Kanehisa, M. & Goto, S. KEGG: kyoto encyclopedia of genes and genomes. *Nucleic Acids Res.* **28**, 27–30 (2000). 10592173 10.1093/nar/28.1.27 PMC102409

[25] Kanehisa, M., Furumichi, M., Sato, Y., Matsuura, Y. & Ishiguro-Watanabe, M. KEGG: biological systems database as a model of the real world. *Nucleic Acids Res.* **53**, D672–D677 (2025). 39417505 10.1093/nar/gkae909 PMC11701520

[26] Kanehisa, M. Toward understanding the origin and evolution of cellular organisms. *Protein Sci.* **28**, 1947–1951 (2019). 31441146 10.1002/pro.3715 PMC6798127

[27] Akbudak, M. A., Filiz, E., Vatansever, R. & Kontbay, K. Genome-wide identification and expression profiling of ascorbate peroxidase (APX) and glutathione peroxidase (GPX) genes under drought stress in sorghum (Sorghum bicolor L). *J. Plant Growth Regul.* **37**, 925–936 (2018).

[28] Kamruzzaman, M. et al. Pinpointing genomic loci for drought-induced proline and hydrogen peroxide accumulation in bread wheat under field conditions. *BMC Plant Biol.* **22**, 584 (2022). 36513990 10.1186/s12870-022-03943-9 PMC9746221

[29] Zhou, Z. et al. Identification of genomic regions affecting grain peroxidase activity in bread wheat using genome-wide association study. *BMC Plant Biol.* **21**, 523 (2021). 34758752 10.1186/s12870-021-03299-6 PMC8579651

[30] Richardson, K. & Jones, M. C. Why genome-wide associations with cognitive ability measures are probably spurious. *New Ideas Psychol.* **55**, 35–41 (2019).

[31] Sullivan, P. F. Spurious genetic associations. *Biol. Psychiatry*. **61**, 1121–1126 (2007). 17346679 10.1016/j.biopsych.2006.11.010

[32] Clemente, A. et al. Eliminating anti-nutritional plant food proteins: the case of seed protease inhibitors in pea. *PLoS One*. **10**, e0134634 (2015). 26267859 10.1371/journal.pone.0134634 PMC4534040

[33] Schulthess, A. W. et al. The roles of pleiotropy and close linkage as revealed by association mapping of yield and correlated traits of wheat (Triticum aestivum L). *J. Exp. Bot.* **68**, 4089–4101 (2017). 28922760 10.1093/jxb/erx214 PMC5853857

[34] Liu, C. et al. Multi-trait genome-wide association studies reveal novel pleiotropic loci associated with yield and yield-related traits in rice. *J. Integr. Agri. *Advance online publication. 10.1016/j.jia.2024.07.026 (2024).

[35] Kruger, N. J. & Von Schaewen, A. The oxidative pentose phosphate pathway: structure and organisation. *Curr. Opin. Plant. Biol.* **6**, 236–246 (2003). 12753973 10.1016/s1369-5266(03)00039-6

[36] Tang, Q. et al. 6-Phosphogluconate dehydrogenase 2 bridges the OPP and shikimate pathways to enhance aromatic amino acid production in plants. *Sci. China Life Sci.* **67**, 2488–2498 (2024). 10.1007/s11427-024-2567-4 39060614

[37] Herrmann, K. M. & Weaver, L. M. The shikimate pathway. *Annu. Rev. Plant Biol.* **50**, 473–503 (1999). 10.1146/annurev.arplant.50.1.473 15012217

[38] Maeda, H. & Dudareva, N. The shikimate pathway and aromatic amino acid biosynthesis in plants. *Annu. Rev. Plant Biol.* **63**, 73–105 (2012). 22554242 10.1146/annurev-arplant-042811-105439

[39] Dahan, J. et al. Disruption of the Cytochrome c oxidase deficient1 Gene Leads to Cytochrome c Oxidase Depletion and Reorchestrated Respiratory Metabolism in Arabidopsis. *Plant Physiol.* **166**, 1788–1802 (2014). 25301889 10.1104/pp.114.248526 PMC4256860

[40] Mansilla, N., Garcia, L., Gonzalez, D. H. & Welchen, E. AtCOX10, a protein involved in haem o synthesis during cytochrome c oxidase biogenesis, is essential for plant embryogenesis and modulates the progression of senescence. *J. Exp. Bot.* **66**, 6761–6775 (2015). 26246612 10.1093/jxb/erv381

[41] Lv, Q. et al. Wheat E3 ubiquitin ligase TaGW2-6A degrades TaAGPS to affect seed size. *Plant Sci.* **320**, 111274 (2022). 35643616 10.1016/j.plantsci.2022.111274

[42] Xia, T. et al. The Ubiquitin Receptor DA1 Interacts with the E3 Ubiquitin Ligase DA2 to Regulate Seed and Organ Size in Arabidopsis. *Plant. Cell.* **25**, 3347–3359 (2013). 24045020 10.1105/tpc.113.115063 PMC3809536

[43] Popescu, S. C. & Tumer, N. E. Silencing of ribosomal protein L3 genes in N. tabacum reveals coordinate expression and significant alterations in plant growth, development and ribosome biogenesis. *Plant J.* **39**, 29–44 (2004). 15200640 10.1111/j.1365-313X.2004.02109.x

[44] Tian, S. et al. Ribosomal protein NtRPL17 interacts with kinesin-12 family protein NtKRP and functions in the regulation of embryo/seed size and radicle growth. *J. Exp. Bot.* **68**, 5553–5564 (2017). 29045730 10.1093/jxb/erx361 PMC5853406

[45] Weis, B. L., Kovacevic, J., Missbach, S. & Schleiff, E. Plant-Specific Features of Ribosome Biogenesis. *Trends Plant Sci.* **20**, 729–740 (2015). 26459664 10.1016/j.tplants.2015.07.003

[46] Pescador-Dionisio, S. et al. Contribution of the regulatory miR156-SPL9 module to the drought stress response in pigmented potato (Solanum tuberosum L). *Plant Physiol. Biochem.* **217**, 109195 (2024). 39442420 10.1016/j.plaphy.2024.109195

[47] Werner, C., Fasbender, L., Romek, K. M., Yáñez-Serrano, A. M. & Kreuzwieser, J. Heat Waves Change Plant Carbon Allocation Among Primary and Secondary Metabolism Altering CO2 Assimilation, Respiration, and VOC Emissions. *Front. Plant. Sci.* **11**, 1242 (2020). 10.3389/fpls.2020.01242 PMC7456945 32922421

[48] Brown, D. C. W. & Thorpe, T. A. Mitochondrial activity during shoot formation and growth in tobacco callus. *Physiol. Plant.* **54**, 125–130 (1982).

[49] Jia, F. et al. Overexpression of Mitochondrial Phosphate Transporter 3 Severely Hampers Plant Development through Regulating Mitochondrial Function in Arabidopsis. *PLOS ONE*. **10**, e0129717 (2015). 26076137 10.1371/journal.pone.0129717 PMC4468087

[50] van der Merwe, M. J. et al. Tricarboxylic Acid Cycle Activity Regulates Tomato Root Growth via Effects on Secondary Cell Wall Production. *Plant Physiol.* **153**, 611–621 (2010). 20118274 10.1104/pp.109.149047 PMC2879791

[51] Verbančič, J., Lunn, J. E., Stitt, M. & Persson, S. Carbon Supply and the Regulation of Cell Wall Synthesis. *Mol. Plant*. **11**, 75–94 (2018). 29054565 10.1016/j.molp.2017.10.004

[52] Bus, A. et al. Species- and genome-wide dissection of the shoot ionome in *Brassica napus* and its relationship to seedling development. *Front. Plant Sci. ***5, **485, (2014). 10.3389/fpls.2014.00485 PMC4179769 25324847

[53] Wang, P., Zhou, G., Cui, K., Li, Z. & Yu, S. Clustered QTL for source leaf size and yield traits in rice (Oryza sativa L). *Mol. Breeding*. **29**, 99–113 (2012).

[54] Aguilar, M. & Prieto, P. Sequence analysis of wheat subtelomeres reveals a high polymorphism among homoeologous chromosomes. *Plant. Genome*. **13**, e20065 (2020). 33029942 10.1002/tpg2.20065 PMC12806930

[55] Fan, C. et al. The Subtelomere of Oryza sativa Chromosome 3 Short Arm as a Hot Bed of New Gene Origination in Rice. *Mol. Plant*. **1**, 839–850 (2008). 19825586 10.1093/mp/ssn050 PMC2902912

[56] Brown, C. A., Murray, A. W. & Verstrepen, K. J. Rapid Expansion and Functional Divergence of Subtelomeric Gene Families in Yeasts. *Curr. Biol.* **20**, 895–903 (2010). 20471265 10.1016/j.cub.2010.04.027 PMC2877759

[57] Saint-Leandre, B. & Levine, M. T. The Telomere Paradox: Stable Genome Preservation with Rapidly Evolving Proteins. *Trends Genet.* **36**, 232–242 (2020). 32155445 10.1016/j.tig.2020.01.007 PMC7066039

[58] Hesami, M., Naderi, R., Tohidfar, M. & Yoosefzadeh-Najafabadi, M. Development of support vector machine-based model and comparative analysis with artificial neural network for modeling the plant tissue culture procedures: effect of plant growth regulators on somatic embryogenesis of chrysanthemum, as a case study. *Plant. methods*. **16**, 1–15 (2020). 32817755 10.1186/s13007-020-00655-9 PMC7424974

[59] Wu, D. et al. Combining high-throughput micro-CT-RGB phenotyping and genome-wide association study to dissect the genetic architecture of tiller growth in rice. *J. Exp. Bot.* **70**, 545–561 (2019). 30380099 10.1093/jxb/ery373 PMC6322582

[60] Seyum, E. G. et al. Genomic selection in tropical perennial crops and plantation trees: a review. *Mol. Breeding*. **42**, 58 (2022). 10.1007/s11032-022-01326-4 PMC10248687 37313015

[61] Sallam, A., Alqudah, A. M., Baenziger, P. S. & Rasheed, A. Editorial: Genetic validation and its role in crop improvement. *Front. Genet.* **13**, 1078246 (2023). 10.3389/fgene.2022.1078246 PMC9846199 36685961

[62] Crossa, J. et al. Expanding genomic prediction in plant breeding: harnessing big data, machine learning, and advanced software. *Trends Plant Sci.* **30**, 756–774 (2025). 39890501 10.1016/j.tplants.2024.12.009

[63] Motamayor, J. C. et al. Geographic and genetic population differentiation of the Amazonian chocolate tree (Theobroma cacao L). *PloS one*. **3**, e3311 (2008). 18827930 10.1371/journal.pone.0003311 PMC2551746

[64] Ge, S. X., Jung, D. & Yao, R. ShinyGO: a graphical gene-set enrichment tool for animals and plants. *Bioinformatics* **36**, 2628–2629 (2020). 31882993 10.1093/bioinformatics/btz931 PMC7178415

[65] Schwenk, H. & Bengio, Y. Boosting neural networks. *Neural Comput.* **12**, 1869–1887 (2000). 10953242 10.1162/089976600300015178

