# Supplementary material for: A GWAS–machine learning framework reveals protein-synthesis pathway signals for yield in Theobroma cacao after population-structure correction
Source: Sci Rep. 2026 Mar 17;16:13840. doi: 10.1038/s41598-026-42273-w (PMC13129028; doi:10.1038/s41598-026-42273-w)
Supplement: Supplementary file 2 — Supplementary Material 2 [file 41598_2026_42273_MOESM2_ESM.docx]

**Supplementary Figures**


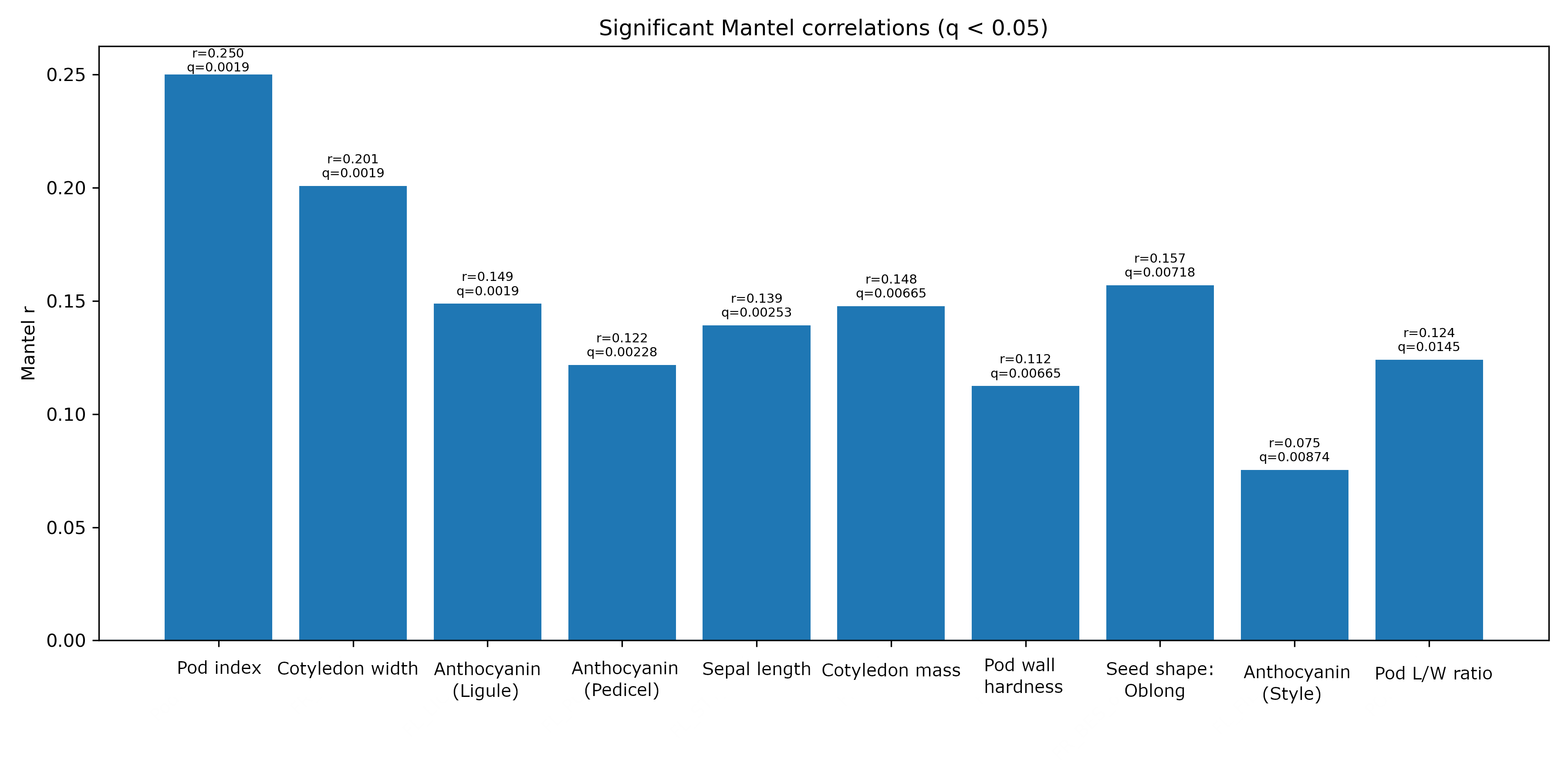


**Fig. S1**

Bars show the Mantel correlation coefficient (*r*) between genetic distance and pairwise phenotypic difference for each trait. Only traits with *q* < 0.05 are displayed; bars are ordered by *q* and then *r*. Text above each bar reports *r* and *q*. Positive *r* indicates that more distantly related accessions tend to differ more in the trait. The categorical label “Acc Group” was excluded a priori.

**­­**


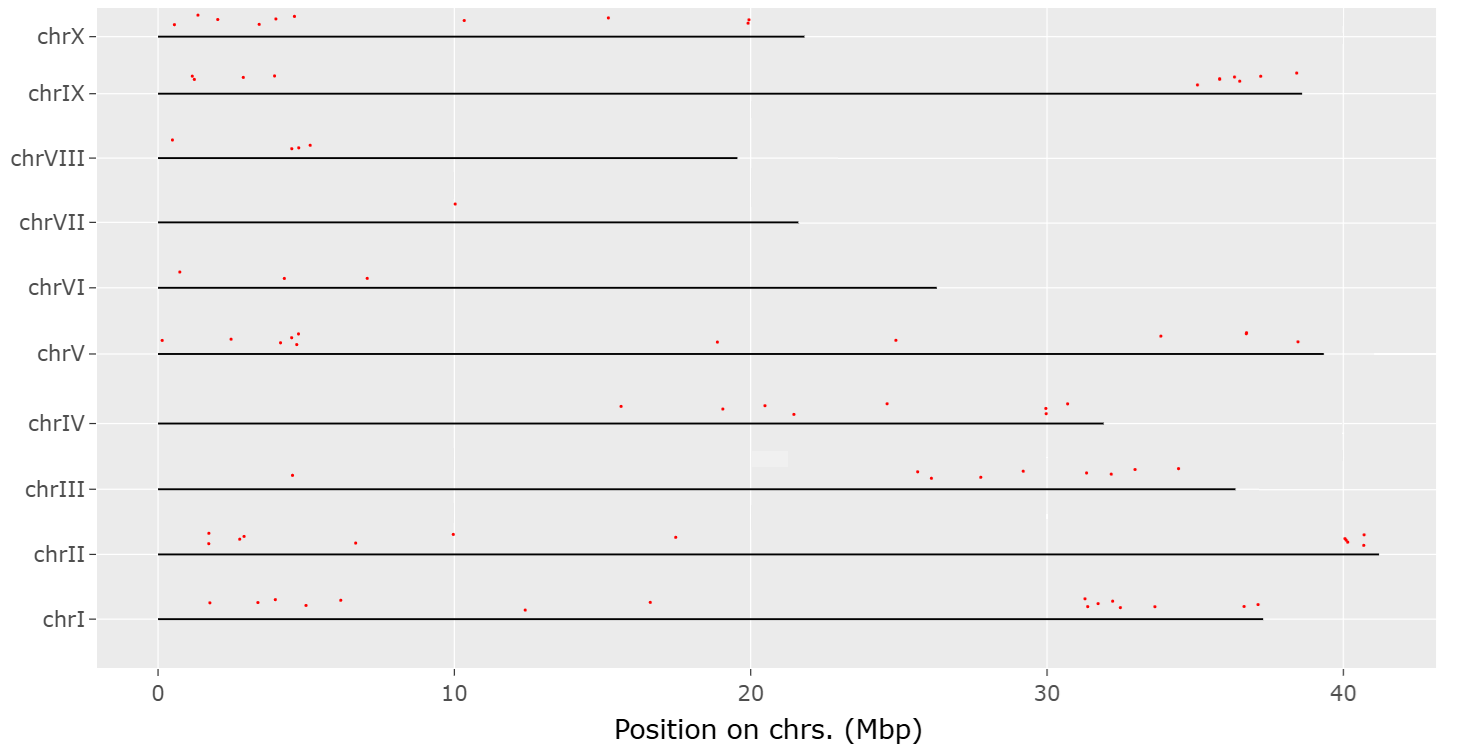
**Fig. S2**

Genomic hotspot map of top associations across all traits. The plot shows the physical locations (Mbp) of the top three top-ranked SNPs for each of the 27 traits analyzed. Clear clusters, or "hotspots," of associated loci are visible on several chromosomes. A complete list of all SNPs shown, their associated traits, and putative candidate genes can be found in Supplementary Data 1.


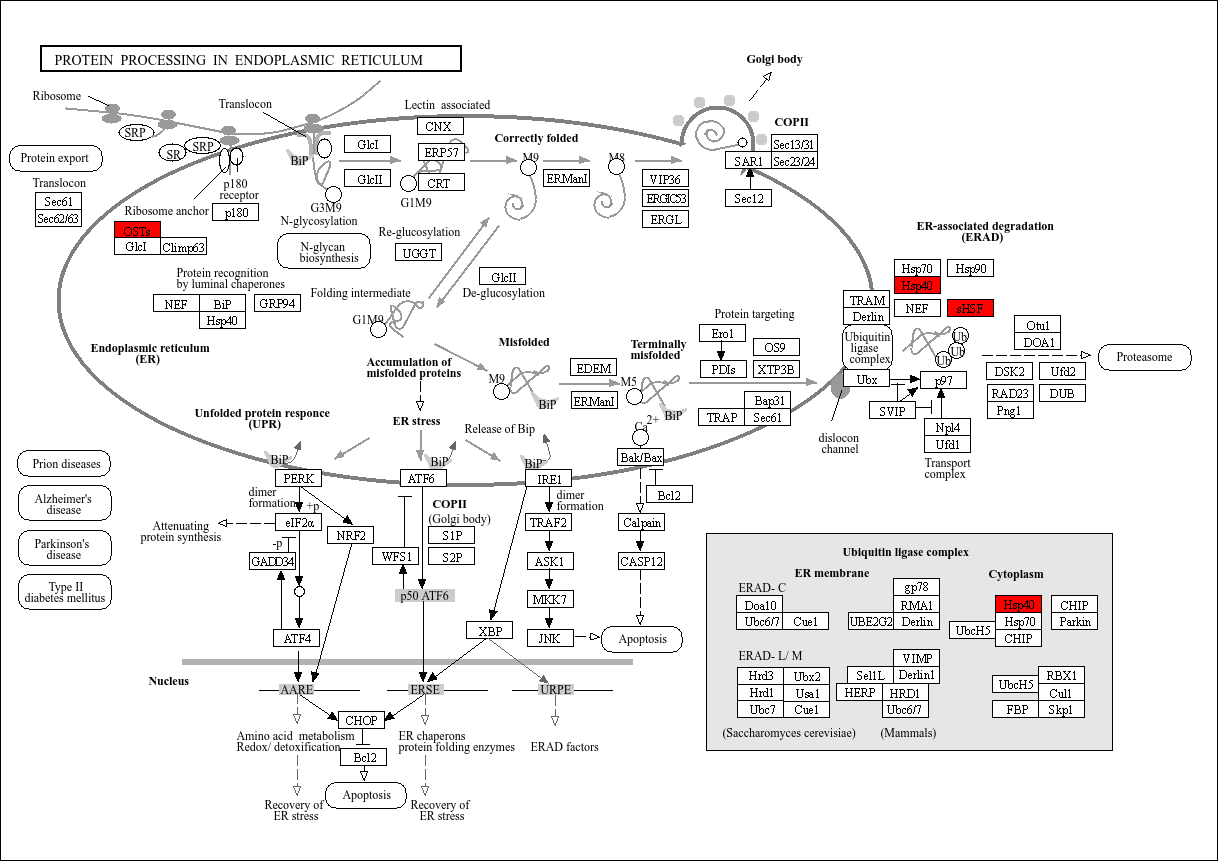


**Fig. S3**

KEGG pathway for protein processing in the endoplasmic reticulum. This pathway was enriched in the uncorrected GWAS for the pod index trait. The analysis identified several key genes within this pathway (highlighted in red), including components of the N-glycosylation machinery (*OST4*) and molecular chaperones involved in protein folding and the ER-associated degradation (ERAD) pathway (*Hsp90*, *Hsp40*). This suggests that protein quality control is a major component of the broad "vigor" signal detected in the naïve analysis. The KEGG pathway map file was obtained from the KEGG database (https://www.kegg.jp/kegg/kegg.html).


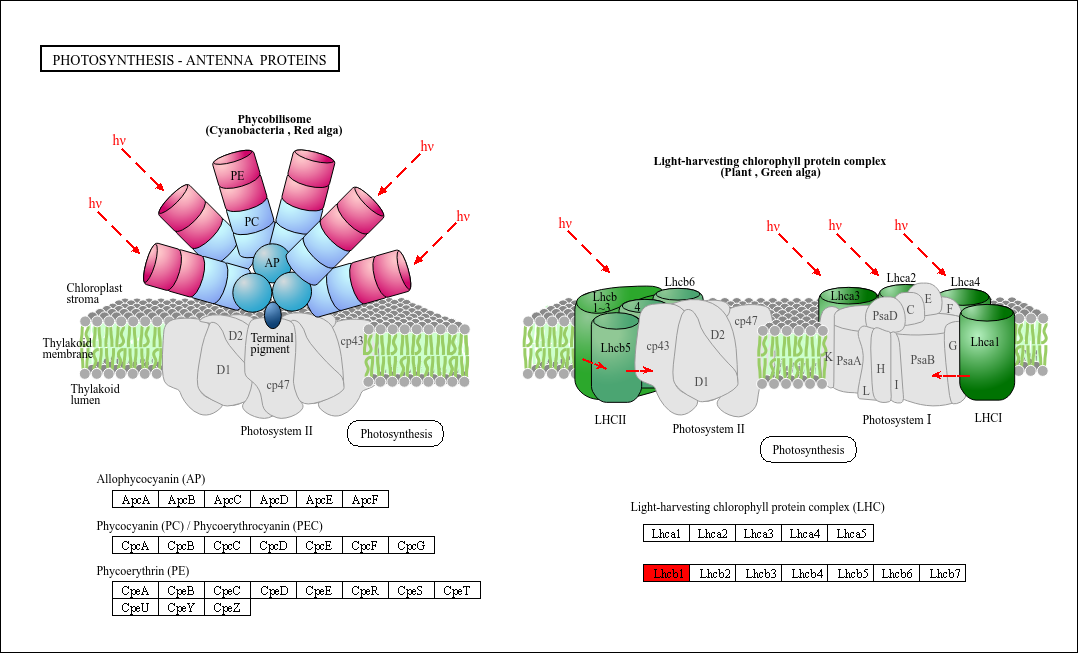


**FIG. S4**

KEGG pathway for photosynthesis - antenna proteins. This pathway was identified as highly enriched in the analysis of the pod index trait for both the naïve (uncorrected) model and the structured association (corrected) model, which highlighted a key gene, *Lhcb1* (highlighted in red), encoding a major light-harvesting chlorophyll-binding protein of photosystem II. The consistent significance of this pathway across both analytical models suggests that the efficiency of light capture is a robust component of the genetic architecture for yield-related traits in this cacao collection. The KEGG pathway map file was obtained from the KEGG database (https://www.kegg.jp/kegg/kegg.html).


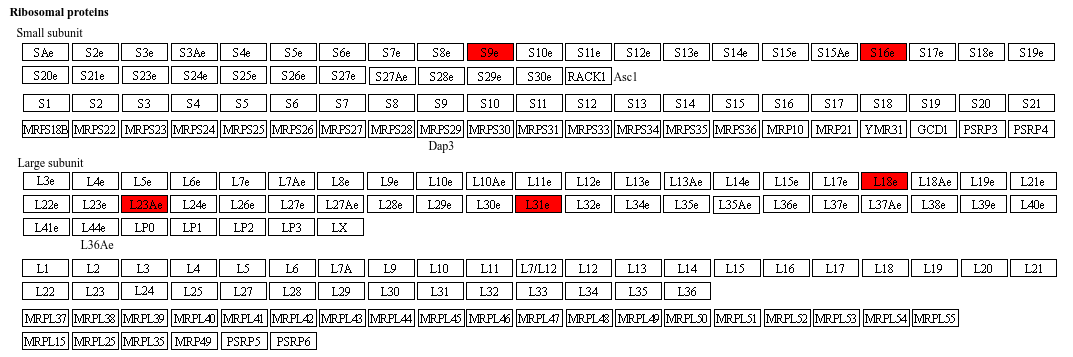


**Fig. S5**

KEGG pathway for the ribosome. This pathway was highly enriched in the structured association (corrected) GWAS for the pod index trait. The analysis identified multiple genes encoding specific ribosomal proteins (highlighted in red), including components of the small subunit (S9e, S16e) and the large subunit (L23Ae, L31e, L18e). This finding is consistent with candidate summaries implicating the protein-synthesis machinery after population-structure correction in this panel. The KEGG pathway map file was obtained from the KEGG database (https://www.kegg.jp/kegg/kegg.html).


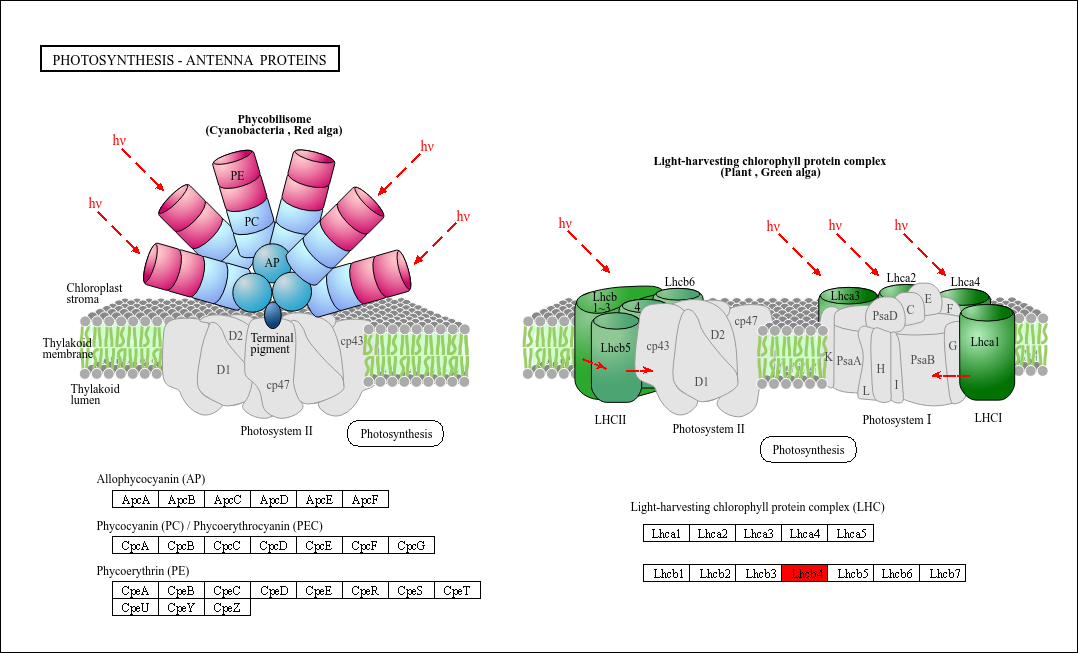


**Fig. S6**

KEGG pathway for photosynthesis - antenna proteins. The pathway was identified as highly enriched in the corrected, combined-trait analysis for the "color & pigmentation" group. The analysis highlighted the gene *Lhcb4* (highlighted in red), which encodes a key component of the light-harvesting chlorophyll protein complex. This finding suggests a specific link between the machinery of light capture and the energy-intensive process of producing the pigments responsible for color. The KEGG pathway map file was obtained from the KEGG database (https://www.kegg.jp/kegg/kegg.html).

**Supplementary Tables**

| **Trait** | **Main effect** | **Total effect** | **Importance score** |
| --- | --- | --- | --- |
| Cotyledon mass | 0.4843 | 0.5198 | 6 |
| Cotyledon length | 0.1864 | 0.2133 | 2 |
| Cotyledon length to width ratio | 0.0867 | 0.1101 | 1 |
| Cotyledon width | 0.04815 | 0.06895 | 0 |

**Table S1**

Most important traits for wet bean mass prediction based on the Neural Boosted model analysis. Relative importance of the top four predictor traits as determined by the Neural Networks. Feature importance was assessed using a 5-fold cross-validation procedure on the full dataset of 346 cacao accessions. The table lists each trait, its main effect, total effect, and importance score. The importance score reflects the contribution of each trait to the model's predictive accuracy, with higher scores indicating a stronger influence. Cotyledon mass was identified as the most important predictive variable.

| **Trait** | **Spearman *ρ* (non-zero SNPs)** | **Shared SNPs (non-zero)** | **Top-20 overlap** |
| --- | --- | --- | --- |
| Anthocyanin intensity (filament) | 0.59 | 628 | 0.35 |
| Anthocyanin intensity (ligule) | 0.58 | 629 | 0.35 |
| Anthocyanin intensity (pedicel column) | 0.632 | 627 | 0.3 |
| Basal constriction | 0.663 | 628 | 0.55 |
| Cotyledon color | 0.603 | 627 | 0.25 |
| Cotyledon length | 0.459 | 622 | 0.2 |
| Cotyledon mass | 0.436 | 621 | 0.15 |
| Cotyledon width | 0.458 | 621 | 0.2 |
| Fruit apex form: Acute | 0.56 | 631 | 0.2 |
| Fruit apex form: Attenuate | 0.412 | 651 | 0.4 |
| Fruit apex form: Indented | 0.528 | 643 | 0.35 |
| Fruit apex form: Mammilate | 0.436 | 635 | 0.35 |
| Fruit apex form: Obtuse | 0.671 | 626 | 0.4 |
| Fruit length | 0.678 | 607 | 0.3 |
| Fruit shape: Elliptic | 0.609 | 641 | 0.3 |
| Fruit shape: Obicular | 0.29 | 635 | 0.35 |
| Fruit shape: Oblate | 0.229 | 602 | 0.45 |
| Fruit shape: Oblong | 0.428 | 644 | 0.5 |
| Fruit shape: Obovate | 0.561 | 630 | 0.45 |
| Fruit surface anthocyanin (mature ridges) | 0.624 | 639 | 0.4 |
| Fruit width | 0.55 | 625 | 0.15 |
| Ligule width | 0.612 | 629 | 0.4 |
| Ovule number | 0.585 | 625 | 0.25 |
| Pod index | 0.579 | 622 | 0.45 |
| Pod length-to-width ratio | 0.674 | 622 | 0.5 |
| Pod wall hardness | 0.635 | 624 | 0.3 |
| Primary ridge separation | 0.342 | 518 | 0.55 |
| Ridge disposition | 0.298 | 619 | 0.35 |
| Cotyledon length-to-width ratio | 0.534 | 616 | 0.2 |
| Seed number | 0.547 | 630 | 0.35 |
| Seed shape: Elliptic | 0.514 | 628 | 0.35 |
| Seed shape: Oblate | 0.537 | 634 | 0.6 |
| Seed shape: Oblong | 0.392 | 634 | 0.3 |
| Sepal length | 0.515 | 624 | 0.15 |
| Style length | 0.546 | 634 | 0.15 |
| Surface texture | 0.632 | 627 | 0.5 |
| Total wet bean mass | 0.516 | 633 | 0.25 |

**Table S2.**

Fixed vs. unfixed concordance in SNP-importance rankings across traits.

For each trait with matched fixed and unfixed outputs, we report (i) the top-20 overlap fraction and (ii) Spearman rank correlation of SNP “Portion” scores computed over SNPs with non-zero importance in either model (Portion > 0 in fixed or unfixed), along with the number of shared non-zero SNPs.
